# Reaction Pathway Differentiation Enabled Fingerprinting Signal for Single Nucleotide Variant Detection

**DOI:** 10.1002/advs.202412680

**Published:** 2025-02-04

**Authors:** Huixiao Yang, Linghao Zhang, Xinmiao Kang, Yunpei Si, Ping Song, Xin Su

**Affiliations:** ^1^ State Key Laboratory of Organic‐Inorganic Composites Beijing Key Laboratory of Bioprocess Beijing Advanced Innovation Center for Soft Matter Science and Engineering College of Life Science and Technology Beijing University of Chemical Technology Beijing 100029 China; ^2^ School of Biomedical Engineering Zhangjiang Institute for Advanced Study and National Center for Translational Medicine Shanghai Jiao Tong University Shanghai 200240 China; ^3^ State Key Laboratory of Natural and Biomimetic Drugs Peking University Beijing 100191 China

**Keywords:** classification, DNA reaction network, kinetics, machine learning, single‐nucleotide variant detection, variant allele frequency

## Abstract

Accurate identification of single‐nucleotide variants (SNVs) is paramount for disease diagnosis. Despite the facile design of DNA hybridization probes, their limited specificity poses challenges in clinical applications. Here, a differential reaction pathway probe (DRPP) based on a dynamic DNA reaction network is presented. DRPP leverages differences in reaction intermediate concentrations between SNV and WT groups, directing them into distinct reaction pathways. This generates a strong pulse‐like signal for SNV and a weak unidirectional increase signal for wild‐type (WT). Through the application of machine learning to fluorescence kinetic data analysis, the classification of SNV and WT signals is automated with an accuracy of 99.6%, significantly exceeding the 80.7% accuracy of conventional methods. Additionally, sensitivity for variant allele frequency (VAF) is enhanced down to 0.1%, representing a ten‐fold improvement over conventional approaches. DRPP accurately identified *D614G* and *N501Y* SNVs in the *S* gene of SARS‐CoV‐2 variants in patient swab samples with accuracy over 99% (n = 82). It determined the VAF of ovarian cancer‐related mutations *KRAS‐G12R*, *NRAS‐G12C*, and *BRAF‐V600E* in both tissue and blood samples (n = 77), discriminating cancer patients and healthy individuals with significant difference (*p* < 0.001). The potential integration of DRPP into clinical diagnostics, along with rapid amplification techniques, holds promise for early disease diagnostics and personalized diagnostics.

## Graphical abstract

The differential reaction pathway probe is presented, which utilizes a dynamic DNA reaction network to separate single‐nucleotide variants (SNVs) and wild‐type (WT) into distinct reaction pathways, generating unique fingerprinting kinetics pulse‐like for SNVs and weak unidirectional signals for WT. High accuracy is achieved for SNV detection in clinical samples including SARS‐CoV‐2 patient swab samples and ovarian cancer patient tissue/blood samples.

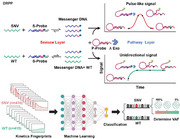

## 1 Introduction

Single‐nucleotide variants (SNVs) carry significant information serving as promising disease indicators for diagnostics and treatment.^[^ ^1^ ^]^ In early disease stages, variant allele frequency (VAF) of SNVs are normally low.^[^ ^1^, ^2^ ^]^ Moreover, regular and widespread testing plays a crucial role in preventing and managing infectious disease outbreaks, exemplified by the SARS‐CoV‐2 pandemic. Given the rapid evolution typical of RNA viruses, numerous mutations in the genome have led to the emergence of variants such as Alpha, Delta, and Omicron.^[^ ^3^ ^]^ The emergence of new mutations may present distinctive features like heightened infectivity, increased toxicity, and enhanced resistance to treatment. This motivates the development of sensitive SNV detection methods.

Presently, the primary methods for detecting SNV heavily rely on next‐generation sequencing (NGS), polymerase chain reaction (PCR), and DNA hybridization probes.^[^ ^4^ ^]^ However, NGS demands a substantial amount of DNA, entails high equipment costs, and involves extended detection cycles. The identification of low‐frequency mutations with NGS necessitates an exceptionally deep sequencing depth.^[^ ^5^ ^]^ PCR‐based approaches lack sufficient sensitivity for all SNV types, leading to inaccurate identifications. Although droplet digital PCR (ddPCR) can detect low abundance, it is hindered by the high costs of reagents and instruments.^[^ ^6^ ^]^ In contrast, DNA hybridization molecular probes (e.g., toehold‐based probes) have recently gained prominence due to their adaptable design, cost‐effectiveness, and rapid sample‐to‐result turnaround. These methods leverage thermodynamic or kinetic differences in the hybridization between SNV/WT and molecular probes, offering a unique solution for distinguishing SNV.^[^ ^7^ ^]^ Performance can be further improved by redesigning the probe structures,^[^ ^8^ ^]^ selectively increasing the abundance of SNV,^[^ ^9^ ^]^ and integrating them with mismatch‐sensitive enzymes.^[^ ^10^ ^]^ Despite the progress in SNV identification through molecular probe technologies, a challenge persists wherein SNV and WT sequences often follow the shared reaction pathways, resulting in limited discrimination capability.^[^ ^11^ ^]^ Moreover, low‐frequency SNV is difficult to detect.^[^ ^12^ ^]^

Dynamic DNA molecular reaction networks refer to DNA circuits or cascades established through thermodynamically driven strand displacement reactions and specific enzymatic reactions. With functions like logical computation, molecular response, and signal amplification, these networks hold great promise for applications in biosensing, biomimetic materials, and drug delivery.^[^ ^12^, ^13^ ^]^ These molecular reaction networks not only demonstrate the ability to manipulate DNA reactions programmatically but also generate unique kinetics signals, such as pulse‐like signals and clock signals.^[^ ^14^ ^]^ Machine learning algorithms can extract valuable information from kinetics data to achieve accurate predictions.^[^ ^15^ ^]^

In this study, we presented a SNV detection system built upon a dynamic DNA reaction network, introducing distinct reaction pathways for WT and SNV sequences. The dynamic reaction network comprised two layers: the Seesaw layer and the Pathway layer. In the Seesaw layer, the reaction resulted in disparate consumption rates between SNV and WT sequences, leading to an instantaneous concentration difference in intermediate products. Simultaneously, in the Pathway layer, SNV and WT sequences followed divergent paths, producing unique kinetic fingerprinting signals. Through the application of machine learning to fluorescence kinetic data, we successfully automated the classification of SNV and wild‐type (WT) signals, achieving a 99.6% accuracy—significantly higher than the 80.7% accuracy of conventional toehold‐based methods. Additionally, we enhanced sensitivity for variant allele frequency (VAF) detection down to 0.1%, representing a 10‐fold improvement over traditional approaches. DRPP identified SNVs *D614G* and *N501Y* in the *S* gene of SARS‐CoV‐2 variants in clinical swab samples with accuracy of 99.1% and 100%. Our method also determined the VAF of ovarian cancer‐related sites (*KRAS‐G12R*, *NRAS‐G12C*, and *BRAF‐V600E*) in clinical tissue and blood samples, enabling the discrimination between ovarian cancer patients and healthy volunteers. The amalgamation of featured fingerprinting signals derived from DRPP with high‐confidence SNV identification through machine learning significantly enhances the precision of SNV detection.

## 2 Results and Discussion

### 2.1 Differential Reaction Pathway Probe (DRPP) and its Procedure for SNV Detection

**Figure 1:**
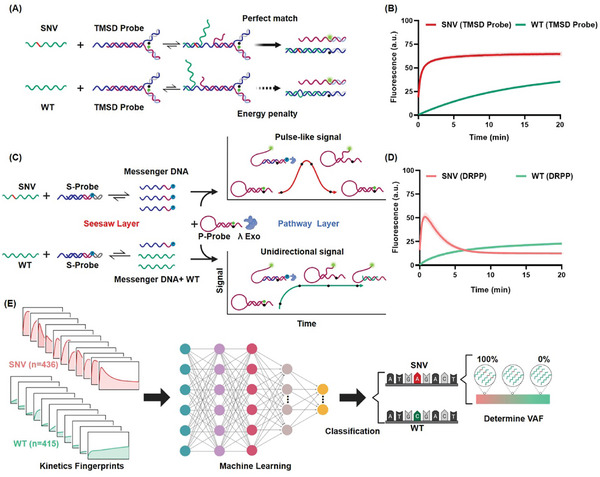
Working principles and signal characterization of SRPP and DRPP for SNV Detection. A) Schematic of SNV detection by SRPP (TMSD probe). Both SNV and WT initiate the strand displacement reactions with the TMSD probe. The binding of WT with the TMSD probe lead to an energy penalty due to a single‐base mismatch, resulting in slower reaction rates. B) Fluorescence kinetics of TMSD probes reacting with SNV/WT (DNA sequences in Table S4, Supporting Information). Data are mean±S.D. (n = 3 independent experiments). C) Schematic of SNV detection by DRPP. The reactions in “seesaw layer” and “pathway layer” are shown. The DNA species are shown in the time‐dependent signal curve. D) Kinetics of DRRP for SNV/WT. Data are mean ± S.D. (n = 3 independent experiments). E) Procedure of SNV identification and VAF determination using machine learning‐based DRPP fluorescence kinetics analysis.

Conventional DNA hybridization probes utilized for discriminating SNVs primarily rely on differences in the thermodynamics and kinetics of reactions involving SNVs and WT sequences upon binding to the probe. Both SNV and WT undergo shared reactions with the probe, thereby termed the Shared reaction pathway probe (SRPP). The Toehold‐Mediated Strand Displacement (TMSD) probe represents a typical SRPP, where the target nucleic acid binds to the toehold region of the probe, triggering branch migration and ultimately releasing the fluorescent strand (**Figure** **1**A). The TMSD probe effectively utilizes thermodynamic energy differences to discriminate between SNV and WT, relying on variations in the strand displacement reaction rate (Figure 1B).^[^ ^16^ ^]^ We conducted reaction kinetics simulations for TMSD probes responding to different concentrations of SNV and WT and found that SNV could be discriminated from WT only within a certain concentration range based on signal intensity (Figure S1, Supporting Information). In the presence of a high concentration of WT background signal, detecting SNV becomes challenging for the TMSD probe.

**Figure 2:**
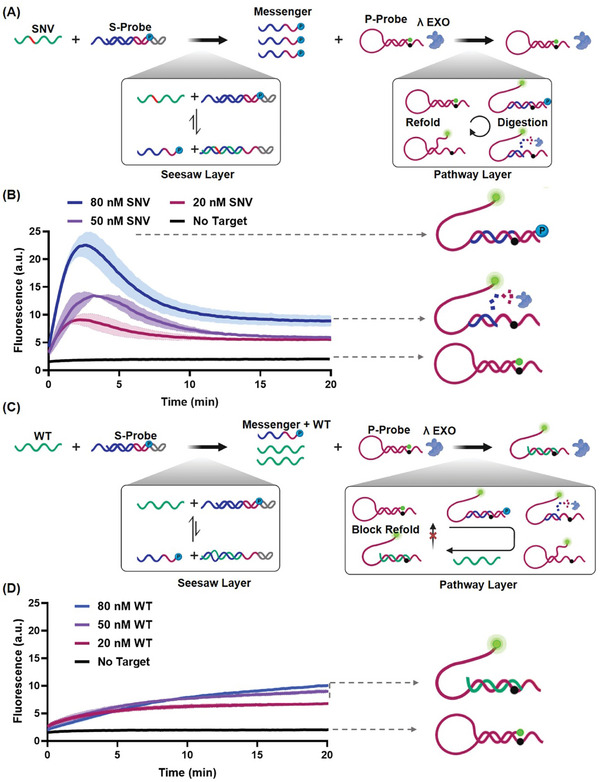
Fluorescence kinetics analysis of SNV and WT detection by DRPP. A) Schematic of the reaction of SNV and DRPP; the SNV binding to S‐Probe initiated a strand displacement reaction driven by free energy, leading to the consumption of a significant portion of SNV species. This process generated a substantial concentration of messenger DNA. Serving as a fuel molecule, the messenger DNA entered the pathway layer, triggering a “fuel dissipation” reaction, and was ultimately consumed, resulting in a pulse‐like fluorescence kinetics signal. B) Fluorescence kinetics of DRPP with different SNV concentrations. Nucleic acid species are marked. Data are mean±S.D. (n = 3 independent experiments). C) Schematic of the reaction of WT and DRPP, the WT sequences initiated a strand displacement reaction with S‐Probe. Mismatch prevented the consumption of most WT species, resulting in a low concentration of messenger DNA. This led to the simultaneous entry of low messenger DNA concentration and high WT concentration into the pathway layer. The messenger DNA first reacted with the P‐Probe, and upon digestion, the WT bound to the opened P‐Probe, blocking its re‐folding. D) Fluorescence kinetics of DRPP with different SNV concentrations. Data are mean±S.D. (n = 3 independent experiments). Nucleic acid species are marked. The forward toehold and reverse toehold of S‐Probe were 7 and 5 nt, respectively. The mismatch site was located at the 7 nt of the 3′ end of the forward toehold of S‐Probe. All reactions were performed at 37 °C in DRPP buffer. All nucleic acid species in DRPP were 100 nm, and λ Exo was 50 U mL^−1^. Detailed information regarding the hybridization regions of the SNV/WT, P‐Probe, and messenger DNA is shown in Figure S5 (Supporting Information).

To address the abovementioned challenges, we devised a dynamic DNA reaction network that directs SNV and WT sequences into distinct reaction pathways, producing signals with distinct shapes. As illustrated in Figure 1C, DRPP comprises two layers. In the first layer “seesaw layer,” we employed the TMSD probe as a control valve for the rate of strand displacement in SNV and WT sequences. Both SNV and WT initiated strand displacement reactions but with varying kinetics. This kinetic dissimilarity leads to the difference of nucleic species over a defined period. SNV undergoes extensive consumption, yielding a high concentration of messenger DNA, while WT was only partially consumed, resulting in a low concentration of messenger DNA. We used a hairpin probe and λ Exo in the second layer, referred to as the “pathway layer,” in which SNV and WT diverged into different reaction pathways. SNV efficiently triggered a “fuel dissipation” reaction (for details see **Figure** **2**A), manifesting as a pulse‐like signal, while WT induced limited hairpin opening (for details see Figure 2C), leading to a unidirectional signal increase. The distinction between SNV and WT is based on the shape of the kinetic data (Figure 1D). Using experimental kinetics datasets (SNV, n = 436; WT, n = 415), we trained a classification model using machine learning. The trained model can then automatically classify SNVs and WTs across various genes without requiring gene‐specific criteria. For real sample detection, the trained model was directly applied to identify SNVs. VAF can also be determined by the established multiclass model (Figure 1E).

### 2.2 Working Principle and Mechanism Study of DRPP

The seesaw layer of DRPP comprises the S‐Probe. The pathway layer consists of P‐Probe and λ Exonuclease (λ Exo), guiding SNV and WT into distinct reaction pathways. The linkage between the two layers is mediated by messenger DNA.

The reaction of DRPP and SNV is shown in Figure 2A. The binding of SNV to the S‐Probe within the seesaw layer initiated the strand displacement reaction, ultimately releasing a messenger DNA strand with a 5′ phosphorylation modification. The strand displacement reaction between SNV and the S‐Probe occurred due to negative free energy (Figure S2, Supporting Information). This resulted in the consumption of SNV and the generation of high concentration of messenger DNA. The messenger DNA then opened the hairpin P‐Probe in the pathway layer, generating the substrate for λ Exo, leading to the digestion of the messenger DNA strand. The opened P‐Probe was then refolded, driven by intramolecular hydrogen bonds. The reaction occurring in the pathway layer represents a typical fuel dissipation reaction. In this process, messenger DNA served as the fuel to shift equilibrium to a high‐energy transient state. Upon the fuel was consumed, the system returned to the equilibrium state. We labeled the 3′ end of the P‐Probe with quencher BHQ1 and the 5′ end with fluorophore FAM. When the P‐Probe was opened by the messenger DNA, the fluorescence signal increased, and when the P‐Probe was refolded, the fluorescence signal decreased. As shown in Figure 2B, pulse‐like fluorescence signals were generated in the SNV and DRPP reactions, as expected. Within the initial 2 min, the fluorescence signal was observed to ascend to its peak, followed by an apparent decline within the subsequent 18 min. A significant pulse‐like pattern was observable within 20 min (Figure 2B). Importantly, the shape of the pulse‐like curve remained unaltered by the concentration of SNV (Figure 2B; Figure S3, Supporting Information). The WT sequence incurred a thermodynamic energy penalty when binding to the S‐Probe in the seesaw layer due to a single‐base mismatch. In the S‐Probe design, a reverse toehold was introduced to enhance the reverse reaction rate, leading to positive free energy in the strand displacement reaction between WT and the S‐Probe (Figure S2, Supporting Information). This resulted in limited consumption of WT. Consequently, the concurrent presence of WT and the messenger DNA strand in the system prompted their involvement in the reaction within the pathway layer. Similarly, the messenger DNA triggered the opening of the hairpin P‐Probe. Subsequently, λ Exo digested the messenger DNA, creating the opened P‐Probe. In contrast to the scenarios of SNV, where S‐Probe consumed a significant portion of SNV in a short time, the WT system harbored WT with high concentration. Upon the digestion of messenger DNA, some of the unconsumed WT hybridized with the opened P‐Probe to form the WT‐P‐Probe complex. Due to the absence of a 5′ phosphorylation of WT, this complex resisted digestion by λ Exo, obstructing the refolding of the opened P‐Probe (Figure 2C). Under these conditions, the expected outcome yielded a unidirectional increase in signal without generating a pulse‐like curve, even over a 12 h period (Figure S4, Supporting Information). As anticipated, the system consistently exhibited a unidirectional fluorescence rise curve regardless of changes in concentration (Figure 2D; Figure S3, Supporting Information).

In addition, DRPP displayed no signal leakage in the absence of a target (Figures 2B,D). Gel electrophoresis was also used to characterize the reaction of SNV/WT with DRPP. As shown in Figure S6A (Supporting Information), lanes 5 and 6 indicated that SNV and WT triggered different degrees of strand displacement of S‐Probe in the seesaw layer, and lane 8 indicated that messenger DNA can bind with P‐Probe through strand displacement. Notably, the messenger DNA in the messenger‐P‐Probe complex in lane 9 underwent digestion by λ Exo and subsequently refolded to form the hairpin P‐Probe. Furthermore, λ Exo did not induce structural changes in the S‐Probe and P‐Probe (Figure S6B, Supporting Information). The hairpin P‐Probe's two bases did not initiate strand displacement with WT (Figure S5, Supporting Information), as verified by gel electrophoresis (Figure S6C, Supporting Information). A kinetics simulation of DRPP was performed. The details of all reactions for SNV and WT are shown in Supporting note. We determined some important reaction constants in DRPP. The kinetics constants for the strand displacement of S‐Probe and P‐Probe were determined by fitting the experimental data shown in Figures S1 and S7 (Supporting Information), and the reaction kinetics constant for λ Exo digestion was determined through the data in Figure S8 (Supporting Information) (for detailed procedure, see Supporting Note, Supporting Information). Based on the measured and presumptive constants, the time‐dependent fluorescent species concentration was simulated, showing a time‐dependent behavior. (Figure S9, Supporting Information). Consistent with the experimental results, the kinetics simulation revealed that SNV yielded a pulse‐like signal, while WT generated a unidirectional increase signal. Also, the mode of the signal was independent of SNV/WT concentration.

**Figure 3:**
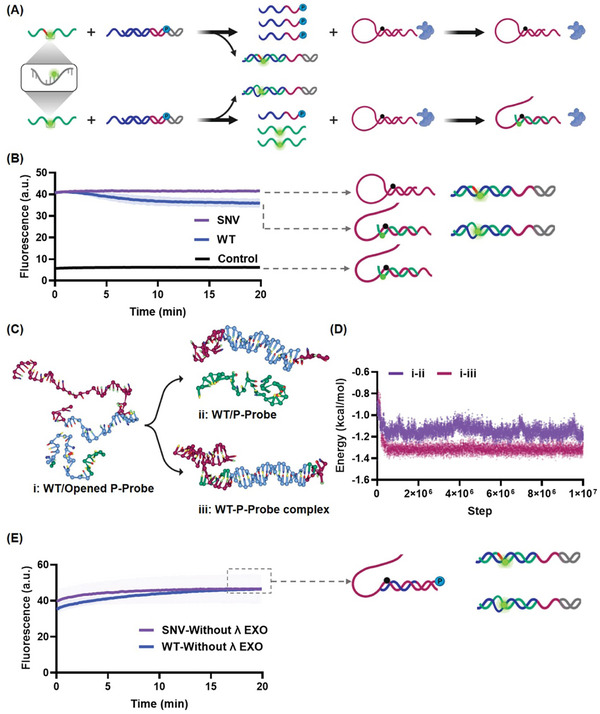
Investigation of the reaction mechanism of DRPP. A) Validation of differential reaction pathways by fluorescence assay. Both SNV and WT were labeled fluorophore, and the P‐Probe was labeled with quencher. Other conditions of DRPP are the same as those in Figure 2A,C. B) Fluorescence kinetics of Figure 3A. WT induced irreversible fluorescence decrease by forming WT‐P‐Probe complex, while SNV did not react with P‐Probe maintaining the high fluorescence. Data are mean±S.D. (n = 3 independent experiments). C) Structures of WT and P‐Probe and possible products presented by coarse‐grained model. The green DNA strand represents WT, the purple DNA strand represents P‐Probe, and the blue is the region of interest for energy and root mean square fluctuation study. D) The coarse‐grained model simulated the energy landscape of the reaction process between WT and opened P‐Probe. E) Fluorescence kinetics from the reactions of panel A in the absence of λ Exo. Data are mean±S.D. (n = 3 independent experiments). The forward toehold and reverse toehold of S‐Probe were 7 and 5 nt, respectively. The mismatch site was located at the 7 nt of the 3′ end of the forward toehold of S‐Probe. All reactions were performed at 37 °C in DRPP buffer. All nucleic acid species in DRPP were 100 nm, and λ Exo was 50 U mL^−1^.

To experimentally validate the differential reaction pathways, we labeled SNV/WT and P‐Probe by fluorophore and quencher, respectively, to monitor the formation of the SNV/WT‐P‐Probe complex (**Figure** **3**A). In Figure 3B, no fluorescence decrease was found SNV group, indicating the absence of SNV‐P‐Probe complex. Conversely, WT showed observable decrease in signal, suggesting the formation of WT‐P‐Probe complex, owing to the generation of this complex. It is noteworthy that only 15% P‐Probe involved in the reaction of WT within 20 min. WT primarily remained in a free state within the assay timeframe. Therefore, we further studied the interaction of WT with opened P‐Probe by molecule dynamic simulation using the coarse‐grained model.^[^ ^17^ ^]^ The possible reaction between WT and opened P‐Probe are shown in Figure 3C: i) WT and opened P‐Probe, ii) the refolded P‐Probe and free WT, and iii) WT‐P‐Probe complex. The coarse‐grained model simulation revealed that the energy followed the order: i>ii>iii (Figure S10A, Supporting Information). The assessment of energy alteration reveals a more pronounced decrease of 0.15 kcal mol^−1^ during the transition from state i to iii, in contrast to the shift from i to ii. This points to a greater likelihood of favorability for the transformation from i to iii (Figure 3D). Upon examining the root mean square fluctuation, the iii state displayed a narrower distribution than the ii state, implying higher stability (Figure S10B, Supporting Information). Thus, the simulation explained the formation of the critical species WT‐P‐Probe complex. In the absence of λ Exo, there was no significant fluorescence change in the SNV and WT groups (Figure 3E). SNV and WT underwent the shared reaction pathway, the P‐Probe binds to the generated messenger DNA, preventing the formation of SNV‐P‐Probe or WT‐P‐Probe complexes. Based on the above experimental and simulation results, the proposed reaction pathways were validated.

### 2.3 Design Flexibility and Broad Reaction Condition Tolerance of DRPP

Conventional molecular probes for SNV detection usually require tedious optimization of conditions (e.g., concentration, structure, temperature) to realize the expected performance.^[^ ^12^, ^18^ ^]^ In contrast, DRPP leverages differential reaction pathways to generate unique fingerprinting signals, making it less sensitive to such variations. We tested the structural influence of the S‐Probe in the seesaw layer by varying forward and reverse toehold lengths (5–7 nt). Both consistently produced pulse‐like signals for SNV and unidirectional signals for WT (Figures S11 and S12, Supporting Information). We also examined mismatch positions (Figure S13, Supporting Information), finding that only mismatches at the probe ends failed to generate distinct signals, indicating high design flexibility. Next, we replaced the hairpin P‐Probe with a double‐stranded structure (Figure S14, Supporting Information), which performed similarly, with SNV causing pulse‐like signals and WT generating unidirectional signals (Figure S15, Supporting Information). Gel electrophoresis confirmed correct operation of the double‐stranded P‐Probe (Figure S16, Supporting Information). We also tested the system's performance under varying λ Exo concentrations (10–250 U mL^−1^) and temperatures (25 °C and 37 °C), finding consistent performance across these conditions (Figures S17 and S18, Supporting Information). These results demonstrate that DRPP features a simple and flexible design with broad adaptability to a wide range of reaction conditions. Good DRPP principles: the toehold length should be between 5–7 nt, the mismatch site is better located in the branch migration region, and the enzyme concentration should range from 10 to 50 U mL^−1^.

### 2.4 DRPP Applicability for SNV Detection

Following the validation of DRPP principles and mechanistic analysis, we demonstrated the robust applicability of DRPP. Detection probes for SNV typically require the capability to identify 12 types of mismatches, including A:A, A:G, A:C, T:T, T:G, T:C, G:A, G:T, G:G, C:A, C:T, and C:C (where the preceding and succeeding letters represent the corresponding bases on the target and probe, respectively).^[^ ^19^ ^]^ This is because these 12 mismatches can cover all types of SNVs. We tested DRPP's signals to discriminate these 12 mismatches and the 4 corresponding matches (DNA sequence in Table S5, Supporting Information). As shown in Figure S19A (Supporting Information), all matches generated pulse‐like fluorescence signals, while mismatches exhibited unidirectional fluorescence increases. This result demonstrated that DRPP was sensitive to all mismatch types, all of which can guaranting the discrimination of all types of SNVs. DRPP's capability to detect SNVs was tested by real genes including *L858R*, *G719S*, *T790* m in the *EGFR* gene,^[^ ^20^ ^]^ *G12R* in the *KRAS* gene, and *F354L* in the STK11 gene related to non‐small cell lung cancer,^[^ ^21^ ^]^ *Y200C* in the *TP53* tumor suppressor gene,^[^ ^22^ ^]^ *H1047R* in the *PIK3CA* linked to breast cancer gene,^[^ ^23^ ^]^ *NRAS‐G12C* associated with melanoma gene,^[^ ^24^ ^]^ as well as *N501Y* and *D614G* in SARS‐CoV‐2 *S* gene.^[^ ^25^ ^]^ Figure S19C (Supporting Information) shows the SNV types of the above sites. As expected, 10 SNVs generated pulse‐like kinetics, while WTs exhibited a unidirectional fluorescence decrease (Figure S19B, Supporting Information, DNA sequences in Table S5, Supporting Information). DRPP with double‐stranded P‐Probe exhibited similar results as DRPP with hairpin P‐Probe (Figures S19B and S20, Supporting Information).

### 2.5 DRPP Fluorescence Kinetics Automated Classification by Machine Learning

**Figure 4:**
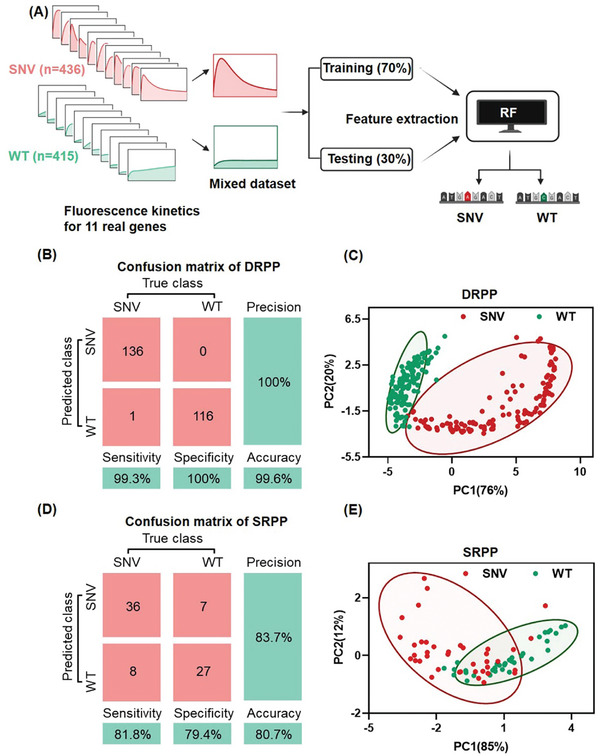
DRPP fluorescence kinetics automated classification based on machine learning. A) The workflow of the machine learning‐based automated classification process for DRPP fluorescence kinetics. The DRPP fluorescence kinetics of the SNVs and WTs in the 11 real genes in Figure 4C were randomly split into a 70% training set and a 30% testing set, and four classifier algorithms were trained for optimization. B) The confusion matrix for DRPP fluorescence kinetics is based on the RF classifier. C) SNV and WT clustering by principal component analysis based on DRPP fluorescence kinetics. D) The confusion matrix for SRPP fluorescence kinetics is based on the RF classifier, with the RF classifier recognized as the optimal model (Figure S22, Supporting Information). E) SNV and WT clustering by principal component analysis based on SRPP fluorescence kinetics.

To increase the result accuracy across genes, we train a classification model for SNV/WT by machine learning based experimental kinetics datasets of SNV and WT. As shown in **Figure** **4**A, the data sets consisted of mixed fluorescence kinetics data originating from SNV (n = 436) and WT (n = 415) of the 11 real genes in Figure S19B (Supporting Information), covering various input concentrations and reaction conditions (temperature, λ Exo concentrations). Random Forest (RF) was chosen for classification.^[^ ^26^ ^]^ The model was verified using split data of the training set of 70% and testing set of 30%. As shown in Figure 4B, the RF classifier provided high accuracy in the confusion matrix. The principal component analysis of fluorescence kinetics for SNV and WT revealing clear clustering of SNV and WT with minimal overlap in the cluster class profiles (Figure 4C and Figure S21, Supporting Information). This can be attributed to the crucial role played by the distinctive fingerprint signals of DRPP, combined with the intelligence and high accuracy of machine learning algorithms. In addition, we used the same method to construct a binary classifier for the fluorescence kinetics of SRPP (SNV n = 145, WT n = 109). The confusion matrix of SRPP showed significant misclassifications in both SNV and WT identification (Figure 4D). The distributions of SNV and WT have a substantial overlap by principal component analysis (Figure 4E). Owing to the same reaction pathway, the shapes of SNV and WT kinetics are nearly identical. As a result, effective distinguishing features extracted by machine learning are limited.

### 2.6 Machine Learning‐Enhanced DRPP for VAF Determination

VAF, reflecting the ratio of SNV and WT in clinical samples, has emerged as a critical parameter for the decision of diagnostics and treatment. Mutant cancer‐related DNA is influenced by non‐tumor DNA backgrounds. VAF varies across different cancer types; for example, the TP53 gene exhibits a high mutation frequency of 90% in ovarian cancer patients compared to only 10% in acute myeloid leukemia patients.^[^ ^27^ ^]^ Additionally, most patients with stage I lung cancer have ctDNA levels of less than 1%.^[^ ^2^, ^28^ ^]^ Identifying low VAF requires extremely deep or targeted sequencing.^[^ ^29^ ^]^ There remains a need for efficient methods to identify and quantify genes with low‐frequency mutation quickly.

**Figure 5:**
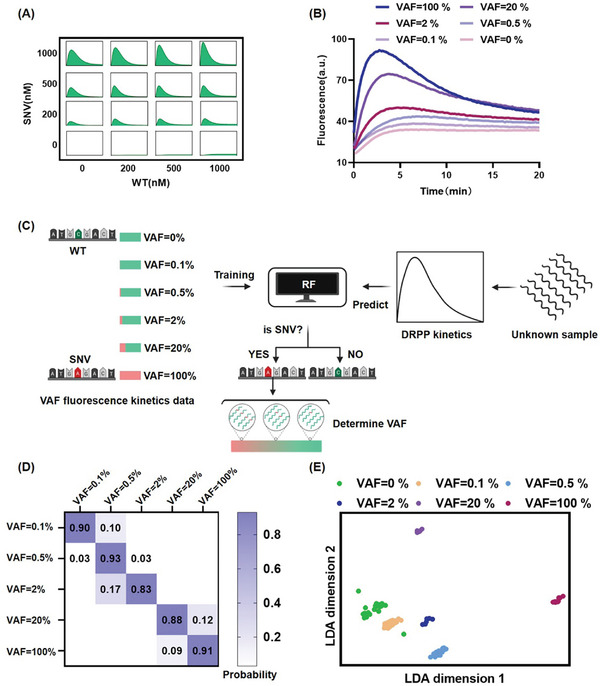
Multi‐classification of DRPP fluorescence kinetics for VAF identification. A) Simulation of DRPP fluorescence kinetics at various SNV/WT ratios. B) Experimental fluorescence kinetics of DRPP for WT (VAF = 0%) and various VAF of 0.1%, 0.5%, 2%, 20%, and 100%. Total concentration of SNV and WT was 500 nm. C) The workflow of automated determination of VAF by machine learning algorithm. SNV was first identified by binary classification, and then VAF was determined by multi‐classification.The binary classification confusion matrix is shown in Figure S22 (Supporting Information). D) The multi‐classification confusion matrix for DRPP fluorescence kinetics of various VAFs based on RF which was recognized as the optimal model. The probability of correct determination is shown. E) Linear discriminant analysis‐based clustering of different VAFs.

With the enhancement of machine learning, DRPP has achieved 99% accuracy for SNV/WT discrimination. And we have verified that DRPP provided concentration‐independent fingerprinting signals. Thus, we anticipated that DRPP could determine VAF. We first obtained the fluorescence kinetics of DRPP at different SNV/WT ratios by kinetics simulation and observed that DRPP was able to display the featured pulse‐like signal in the presence of SNV, while DRPP displayed a unidirectional signal rise only in the presence of WT (**Figure** **5**A). Next, we experimentally examined different VAF samples using DRPP. Consistent with the kinetics simulation results, DRPP exhibited pulse‐like signal when SNVs were present, with varying signal strengths corresponding to different frequencies of SNVs (Figure 5B). This suggests that in mixed SNV and WT samples, the SNV reaction pathway is essentially undisturbed. This can be attributed to WT's low reaction yield (Figure 3B) and slow reaction kinetics (Figure 2B).

These experimental results can be used to train machine learning for automated classification. As shown in Figure 5C, we first utilized a binary classification model for the automated classification of SNV versus WT. The fluorescence kinetics data WT (n = 75) and SNV (VAF = 0.1%–100%, n = 348) of DRPP assay was randomly split into a 70% training set and a 30% test set. As illustrated in Figure S22 (Supporting Information), the resulting confusion matrix demonstrated a classification accuracy of 95%. Subsequently, we developed a multiclass classification model to differentiate between various VAFs within the SNV dataset. The dataset was also randomly divided into a 70% training set and a 30% test set. As demonstrated in Figure 5D, the multiclass classification confusion matrix indicates high accuracy and provides probabilities of sample frequencies, which can be utilized to calculate VAFs between the values. Furthermore, visualizing the linear discriminant analysis of the test set reveals clear sample clustering, with distinct clusters separated by significant distances, highlighting the effectiveness of the multiclass classification approach (Figure 5E). This demonstrates that machine learning‐based multi‐classification of DRPP fluorescence kinetics can accurately determine low‐frequency SNV in a large amount of WT.

### 2.7 SNV Detection in Clinical Samples

To detect SNVs in clinical samples, we first validated the process using plasmids, as shown in Figure S23A (Supporting Information). Plasmids were constructed for KRAS, NRAS, BRAF, and the SARS‐CoV‐2 S gene (Figure S23B, Supporting Information). Human gene plasmids included three mutations (KRAS‐G12R, NRAS‐G12C, BRAF‐V600E), while the SARS‐CoV‐2 plasmid had two mutations (D614G, N501Y) and the conserved S gene sequence. To enable multiplex detection, DRPP probes were modified with HEX, ROX, and FAM for specific mutation sites. Asymmetric PCR amplified target DNA into single‐stranded amplicons (sequences in Table S6, Supporting Information), and DRPP provided fingerprinting signals for automated SNV/WT classification using optimized machine learning. Testing various plasmid combinations (Figure S24, Supporting Information) showed distinct signals for SNVs and WTs, with the binary classification model achieving perfect accuracy (Figure S25A, Supporting Information) and an ROC‐AUC of 1 (Figure S25B, Supporting Information). Principal component analysis confirmed clear separation between SNV and WT clusters without overlap (Figure S25C, Supporting Information).

**Figure 6:**
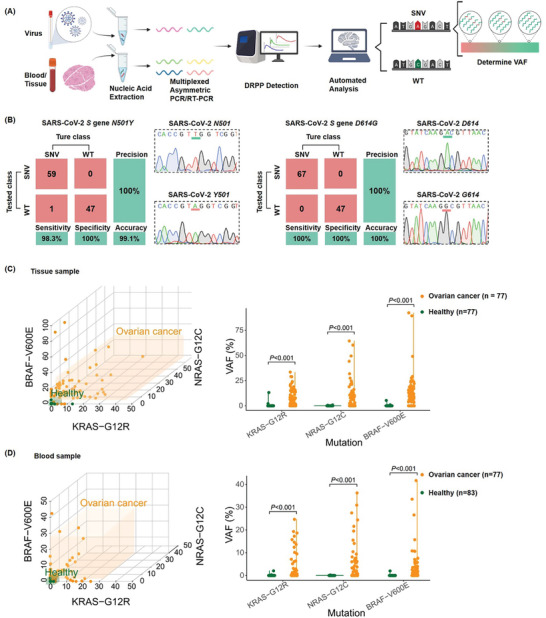
SNV detection in clinical samples. A) Workflow of SNV detection in clinical samples. Nucleic acids were extracted from virus transport medium, patient serum, and tissue. The regions containing SNVs of interest were amplified by asymmetric PCR or asymmetric RT‐PCR to generate single‐stranded amplicons for DRPP detection. Multiplexed DRPP assays were performed to obtain fingerprinting signals for machine learning‐based automated analysis. B) The confusion matrix of DRPP fluorescence kinetics is based on the RF classifier, and representative Sanger sequencing results are shown. C) VAF at three mutation sites (*KRAS G12R*, *BRAF V600E*, and *NRAS G12C*) in ovarian cancer and healthy tissue samples. Orange: tumor samples. Green: healthy samples. *p* < 0.001, calculated by t‐test. D) Variant allele frequency at three mutation sites (*KRAS G12R*, *BRAF V600E*, and *NRAS G12C*) in ovarian cancer and healthy blood samples. Orange: tumor samples. Green: healthy samples. *p* < 0.001 (*t*‐test).

Subsequently, we proceeded to assess SNVs in clinical samples, encompassing swab specimens of SARS‐CoV‐2 wild type and variants, along with blood/tissue samples from ovarian cancer cases. The SARS‐CoV‐2 samples were obtained from the China‐Japan Friendship Hospital, while the ovarian cancer samples originated from the SUN YAT‐SEN University Cancer Center. Viral RNA extraction was isolated from swabs, cell‐free DNA (cfDNA) was extracted from plasma, and genomic DNA was isolated from tissues. The testing protocol mirrored that of plasmid samples, involving asymmetric PCR or RT‐PCR to generate detectable amplicons for multiplexed DRPP assays. DRPP signals were subjected to analysis by machine learning algorithms for automated SNV/WT classification and determination of SNV frequency (**Figure** **6**A).

Our initial focus was on the *D614G* and *N501Y* mutations in the *S* gene of SARS‐CoV‐2, which are recognized as key mutations in variants of concern by the World Health Organization.^[^ ^30^ ^]^ RT‐qPCR of N and orf1ab genes confirmed all samples, with C_t_ values below 35 (Tables S1 and S2, Supporting Information). Sequencing of RT‐PCR products validated the SNVs of *D614G* and *N501Y* in the *S* gene (*D614G* variants n = 60, *N501Y* variants n = 67, WT samples n = 47). Notably, high accuracy and precision were achieved for both *D614G* and *N501Y*, as evidenced by the confusion matrix results. Sensitivity for *D614G* and *N501Y* SNV samples reached 98.3% and 100%, respectively, with 100% specificity for both sites. The representative sequencing results are shown (Figure 6B). Inspired by the success in identifying SNVs in the SARS‐CoV‐2 *S* gene, we extended the use of DRPP to determine VAF in ovarian cancer clinical samples (Table S3, Supporting Information). *KRAS‐G12R*, *NRAS‐G12C*, and *BRAF‐V600E* were selected as potential gene variation biomarkers for ovarian cancer. Paired healthy tissue, cancer tissue, and blood samples from ovarian cancer patients (n = 77) were analyzed, along with 83 blood samples from healthy individuals. Figure 6C,D shows the VAF in all tissue and blood samples. Notably, VAF was calculated as the expected value using machine learning‐based multi‐classification probability. Considering the detection limit of DRPP, VAF <0.1% was classified as WT. The analysis revealed that 78%, 62%, and 78% of cancer tissue samples exhibited *KRAS‐G12R*, *NRAS‐G12C*, and *BRAF‐V600E* mutations, respectively. Correspondingly, 39%, 48%, and 68% of blood samples showed these mutations. Over 95% of healthy tissue and blood samples did not show the mutations. Significant difference was found between cancer and healthy samples for the three sites. The combination of DRPP with machine learning‐based automated classification accurately identified *D614G* and *N501Y* SNVs in the *S* gene of SARS‐CoV‐2 variants, and determined VAF of ovarian cancer‐related sites, allowing for the discrimination of cancer patients and healthy individuals.

## 3 Conclusion

We have developed an innovative SNV detection approach, named DRPP, leveraging the unique kinetics of DNA chemical reaction networks. In contrast to conventional methods relying on signal intensity and shared reactions for both SNV and WT sequences, DRPP introduces distinct reaction pathways for SNV and WT. This results in concentration‐independent fingerprinting kinetics signals, where SNV displays pulse‐like signals, while WT exhibits unidirectional rising signals. DRPP boasts robust performance, simplicity (comprising only four short oligonucleotides and one commercially available enzyme, all components can be pre‐mixed for use), and broad adaptability to various reaction conditions, eliminating the need for intricate optimization. Moreover, DRPP excels in adapting to various clinically relevant mutations. Leveraging machine learning‐based analysis of DRPP kinetics enables automated classification of SNV and WT, accurately determining Variant VAF ranging from 0.1% to 100%. The application of DRPP, coupled with machine learning automation, enables precise identification of specific SNVs, such as *D614G* and *N501Y* in the *S* gene of SARS‐CoV‐2 variants. It also determines VAF for critical ovarian cancer‐related sites, such as *KRAS‐G12R*, *NRAS‐G12C*, and *BRAF‐V600E*, in both tissue and blood cfDNA samples. The sample‐to‐result time is efficiently controlled within 115 min. While ddPCR and NGS have been powerful for SNV detection, they have limitations. ddPCR, despite high sensitivity which can reach <0.1% VAF, suffers from costly instrumentation and reagents, complex workflows, and limited dynamic ranges. NGS, though fundamental for discovering SNVs, has drawbacks in clinical diagnostics, including longer processing times, complex data analysis protocols, and expensive instrumentation and reagents.

Compared to these methods, DRPP emerges as a molecular probe with distinct advantages, offering low cost, rapid sample‐to‐result times, and easily interpretable results. We anticipate that DRPP can be readily deployed for clinical diagnosis, offering a superior alternative in the field of SNV detection.

## 4 Experimental Section

### Reagents and Materials

All oligonucleotides and plasmids used in this work were synthesized by Sangon Biotech (Shanghai) Co., Ltd., and the sequences were listed in Tables S4 and S5 (Supporting Information). All modified oligonucleotides were purified by HPLC, while unmodified oligonucleotides were purified by PAGE. The enzymes and their corresponding buffers were obtained from New England Biolabs (NEB). Other chemicals used in this work were of analytical grade. DNase/RNase‐free deionized water was used in all experiments.

### Kinetics Simulation

The time‐dependent concentration changes in the dissipation reactions computationally simulated by solving the differential equation via Runge–Kutta methods (see supporting information for details regarding the parameter setup).

### Fluorescence Kinetics Assays of the Probes

DNA probes were prepared by mixing the corresponding single strands with equal concentrations (e.g., 1000 nM) in DRPP Buffer (25 mm Tris‐HCl, 50 mm NaCl, 1 mm DTT, 0.1 mm EDTA, 50% Glycerol, pH 8) in 200 µl PCR tubes. The strands were annealed in a PCR thermal cycler from 90 °C to 37 °C at a rate of 1 °C min^−1^, and then λ Exo was added. Upon the addition of target strands, fluorescence was recorded immediately in the corresponding fluorescence channel of a real‐time PCR cycler (Rotor–Gene Q, Qiagen, Germany) at 37 °C using a gain of 4 and a time interval of 5 s.

### Characterization of DRPP Structures and Reactivity by Polyacrylamide Gel Electrophoresis (PAGE)

The products of DRPP reactions were verified by native 15% polyacrylamide (19:1 acrylamide/bisacrylamide) gel electrophoresis. The experiments were performed in Tris/Borate/EDTA buffer (44.5 mm Tris, 44.5 mm Boric acid, 1 mm EDTA, pH 8.0). 5 µl of sample (500 nm) were mixed with 2 µl of loading buffer, and then, the mixture was added to the gel for electrophoresis. All samples were run at 120 V for 60 min at room temperature. After 15 min of staining in SYBR gold (Invitrogen) dissolved in the Tris/Borate/EDTA buffer at pH 8.0, the gel was photographed with a gel imaging system.

### oxDNA Simulation

Simulation of the reaction between WT and opened P‐Probe was operated on oxDNA platform, which was a coarse‐grained model simulation platform.^[^ ^17^ ^]^ The analysis contains two steps: (1) DNA structure construction step and (2) structural movement analysis step. In the case, the initial DNA structures were generated from oxView which provides some simple functions to build DNA structures (https://sulcgroup.github.io/oxdna‐viewer/), and then, two simulation files, “WT.top” and “WT.dat,” could be downloaded for oxDNA simulation. Graphical representations of the generated DNA nanostructure were shown in Figure 3C. Usually, the abovementioned simulation files need to be “relaxed” in oxDNA to produce initial structure files for virtual move Monte Carlo simulation. After each initial structure was generated as expected, the molecular behavior of the DNA structures was simulated by sequence‐dependent virtual move Monte Carlo simulation conditions. During the simulation, the changes in the energy landscape of DNA over time were recorded in “energy.dat,” and the energy landscape changes were plotted as shown in Figure 3D.

### Machine Learning

The machine learning algorithms were implemented in Python using the RF, algorithms from the SKlearn library. The dataset (DRPP kinetics) was split into a training set (70%) and a test set (30%). During the model training process, the grid search method was employed to search for the optimal hyperparameters, and cross‐validation was used to evaluate the performance of different hyperparameter combinations on the test set, with accuracy as the criterion for hyperparameter selection. The machine learning analysis process was identical for both SRPP and DRPP.

### Multiplexed SNV Detection in Plasmid Samples

The plasmids were constructed with the *S* gene in SARS‐CoV‐2 and human genes *KRAS*, *NRAS*, *BRAF*, including mutations and wild‐types. To perform asymmetric PCR, each PCR tube contained 2.5 µL of forward primer (10 µm), 2.5 µL of reverse primer (1 µm), 1 µL of plasmid (1 ng µL^−1^), 25 µL Q5 High‐Fidelity Master Mix (NEB #M0492L), and 19 µL ddH_2_O. PCR procedure (98 °C for 10 s, 55 °C for 30 s, 72 °C for 30 s, 30 cycles) was performed in a PCR thermal cycler. 25 µL PCR products were incubated with 25 µL DRPP mix (100 nm S‐probe, 100 nm P‐Probe, and 50 U mL^−1^ λ Exo) to generate the DRPP kinetics data.

### SARS‐CoV‐2 RNA Extraction from Oropharyngeal Swabs and SNV Detection

Oropharyngeal swab samples of SARS‐CoV‐2 wild type and variants were from China‐Japan friendship hospital. All samples were confirmed as positive based on the C_t_ value (<35) of RT‐qPCR of *orf1ab* and *N* genes (Tables S1 and S2, Supporting Information). The swab samples were introduced into a mixture containing 1 mL lysis buffer and 600 µL ethanol. Subsequently, this mixture was transferred to an adsorption column and centrifuged at 12 000 rpm for 15 s. The elution process yielded the RNA sample, which was collected using RNase‐free water. Then asymmetric PCR was performed as the above procedure, 16 µL PCR products were incubated with 4 µL DRPP mix (final concentration 100 nM S‐probe, 100 nM P‐Probe, and 50 U mL^−1^ λ Exo) to generate the DRPP kinetics data.

### cfDNA Extraction from Blood, Tissue DNA Extraction, and SNV Detection

Clinical paired blood plasmas and tissues were obtained from a cohort of 77 ovarian cancer patients at the SUN YAT‐SEN University Cancer Center (for details see Table S3, Supporting Information), along with 83 blood samples from healthy volunteers. Using a cell‐free blood collection tube, 5 mL of blood was collected from both patient and volunteer groups. Subsequently, the blood samples underwent centrifugation at 1600 g at 4 °C for 10 min to isolate plasma, followed by a second centrifugation at 16,000 g for 10 min at 4 °C to eliminate residual cells.

Cell‐free DNA extraction from 1.5 mL plasma was carried out using the QIAamp Circulating Nucleic Acid Kit (Qiagen, #55114) as per the manufacturer's protocol. The elution volume for each sample was 50 µL in AVE buffer (Qiagen), and the extracted material was stored at −80 °C until needed. Genomic DNAs from frozen tissues were isolated using the Blood & Tissue Kit (Qiagen, #69504) following the provided guidelines. For both cell‐free DNA and tissue genomic DNA, an asymmetric PCR was employed to generate single‐stranded amplicons for DRPP detection. Subsequently, 16 µL of PCR products were incubated with 4 µL of DRPP mix (final concentration: 100 nm S‐probe, 100 nm P‐Probe, and 50 U mL^−1^ λ Exo) to generate the DRPP kinetics data. For the application of VAF detection, higher gain level of the fluorescence detector was used.

### Statistical Analysis

Fluorescence data were presented as the mean ± standard deviation (SD). Kinetic analysis of SRPP and DRPP was conducted using the Random Forest algorithm in machine learning. Clinical sample data were analyzed using R Project for statistical analysis, with a t‐test applied to compare ovarian cancer and healthy samples. Statistical significance was defined as P < 0.05.

## Conflict of Interest

The authors declare no conflict of interest.

## Supporting information



Supporting Information

## Data Availability

The data that support the findings of this study are available from the corresponding author upon reasonable request.

## References

[1] a) A. M. Miller , R. H. Shah , E. I. Pentsova , M. Pourmaleki , S. Briggs , N. Distefano , Y. Zheng , A. Skakodub , S. A. Mehta , C. Campos , Nature 2019, 565, 654; 30675060 10.1038/s41586-019-0882-3 PMC6457907

[2] a) C. Abbosh , N. J. Birkbak , G. A. Wilson , M. Jamal‐Hanjani , T. Constantin , R. Salari , J. Le Quesne , D. A. Moore , S. Veeriah , R. Rosenthal , Nature 2017, 545, 446;28445469

[3] a) S. Kumar , T. S. Thambiraja , K. Karuppanan , G. Subramaniam , J. Med. Virol. 2022, 94, 1641; 34914115 10.1002/jmv.27526

[4] a) S. Tyagi , D. P. Bratu , F. R. Kramer , Nat. Biotechnol. 1998, 16, 49; 9447593 10.1038/nbt0198-49

[5] a) D. Akhoundova , M. A. Rubin , Cancer Cell 2022, 40, 920; 36055231 10.1016/j.ccell.2022.08.011

[6] a) C. M. Hindson , J. R. Chevillet , H. A. Briggs , E. N. Gallichotte , I. K. Ruf , B. J. Hindson , R. L. Vessella , M. Tewari , Nat. Methods 2013, 10, 1003; 23995387 10.1038/nmeth.2633 PMC4118677

[7] W. Tang , W. Zhong , Y. Tan , G. A. Wang , F. Li , Y. Liu , Top. Curr. Chem. 2020, 378, 10. 10.1007/s41061-019-0274-z 31894426

[8] a) Y. Yu , T. Wu , A. Johnson‐Buck , L. Li , X. Su , Biosens. Bioelectron. 2016, 82, 248; 27100949 10.1016/j.bios.2016.03.070

[9] W. Tang , Y. Zhang , J. Wang , Y. Zhao , X. Xu , C. Liu , Y. Liu , X. Zhang , Anal. Chem. 2022, 94, 5838. 35385254 10.1021/acs.analchem.1c05280

[10] a) F. Hong , D. Ma , K. Wu , L. A. Mina , R. C. Luiten , Y. Liu , H. Yan , A. A. Green , Cell 2020, 180, 1018; 32109416 10.1016/j.cell.2020.02.011 PMC7063572

[11] a) H. Hao , Y. Li , B. Yang , S. Lou , Z. Guo , W. Lu , Anal. Chem. 2023, 95, 2893; 36695821 10.1021/acs.analchem.2c04446

[12] a) H. Yu , X. Han , W. Wang , Y. Zhang , L. Xiang , D. Bai , L. Zhang , Z. Weng , K. Lv , L. Song , ACS Nano 2024, 18, 12401; 38701333 10.1021/acsnano.4c01511

[13] a) L. Zhang , H. Zhao , H. Yang , X. Su , Biosens. Bioelectron. 2023, 239, 115622; 37611449 10.1016/j.bios.2023.115622

[14] a) J. Chen , S. Fu , C. Zhang , H. Liu , X. Su , Small 2022, 18, 2108008; 10.1002/smll.202108008 35254723

[15] a) S. Min , B. Lee , S. Yoon , Briefings Bioinf. 2017, 18, 851; 10.1093/bib/bbw068 27473064

[16] D. Y. Zhang , S. X. Chen , P. Yin , Nat. Chem. 2012, 4, 208. 22354435 10.1038/nchem.1246 PMC4238961

[17] R. R. Machinek , T. E. Ouldridge , N. E. Haley , J. Bath , A. J. Turberfield , Nat. Commun. 2014, 5, 5324. 25382214 10.1038/ncomms6324

[18] L. Zhang , J. Chen , M. He , X. Su , Exploration 2022, 2, 20210265. 37324584 10.1002/EXP.20210265 PMC10190925

[19] a) C. Wang , J. Dai , N. Qin , J. Fan , H. Ma , C. Chen , M. An , J. Zhang , C. Yan , Y. Gu , Cancer Cell 2022, 40, 1223; 36113475 10.1016/j.ccell.2022.08.013

[20] S. V. Sharma , D. W. Bell , J. Settleman , D. A. Haber , Nat. Rev. Cancer. 2007, 7, 169. 17318210 10.1038/nrc2088

[21] S. Mueller , T. Engleitner , R. Maresch , M. Zukowska , S. Lange , T. Kaltenbacher , B. r. Konukiewitz , R. Ã−llinger , M. Zwiebel , A. Strong , Nature 2018, 554, 62. 29364867 10.1038/nature25459 PMC6097607

[22] E. H.‐C. Hsiue , K. M. Wright , J. Douglass , M. S. Hwang , B. J. Mog , A. H. Pearlman , S. Paul , S. R. DiNapoli , M. F. Konig , Q. Wang , Science 2021, 371, eabc8697. 33649166 10.1126/science.abc8697 PMC8208645

[23] a) K. M. Vasudevan , D. A. Barbie , M. A. Davies , R. Rabinovsky , C. J. McNear , J. J. Kim , B. T. Hennessy , H. Tseng , P. Pochanard , S. Y. Kim , Cancer Cell 2009, 16, 21; 19573809 10.1016/j.ccr.2009.04.012 PMC2752826

[24] C. Greenman , P. Stephens , R. Smith , G. L. Dalgliesh , C. Hunter , G. Bignell , H. Davies , J. Teague , A. Butler , C. Stevens , Nature 2007, 446, 153. 17344846 10.1038/nature05610 PMC2712719

[25] K. Leung , M. H. Shum , G. M. Leung , T. T. Lam , J. T. Wu , Euro Surveill. 2021, 26, 2002106.33413740

[26] M. I. Jordan , T. M. Mitchell , Science 2015, 349, 255. 26185243 10.1126/science.aaa8415

[27] L. A. Donehower , T. Soussi , A. Korkut , Y. Liu , A. Schultz , M. Cardenas , X. Li , O. Babur , T.‐K. Hsu , O. Lichtarge , Cell Rep. 2019, 28, 1370. 31365877 10.1016/j.celrep.2019.07.001 PMC7546539

[28] A. A. Chaudhuri , J. J. Chabon , A. F. Lovejoy , A. M. Newman , H. Stehr , T. D. Azad , M. S. Khodadoust , M. S. Esfahani , C. L. Liu , L. Zhou , Cancer Discov. 2017, 7, 1394. 28899864 10.1158/2159-8290.CD-17-0716 PMC5895851

[29] a) P. Song , S. X. Chen , Y. H. Yan , A. Pinto , L. Y. Cheng , P. Dai , A. A. Patel , D. Y. Zhang , Nat. Biomed. Eng. 2021, 5, 690; 33941896 10.1038/s41551-021-00713-0 PMC9631981

[30] P. Zhou , X.‐L. Yang , X.‐G. Wang , B. Hu , L. Zhang , W. Zhang , H.‐R. Si , Y. Zhu , B. Li , C.‐L. Huang , Nature 2020, 579, 270. 32015507 10.1038/s41586-020-2012-7 PMC7095418

