# Supplementary material for: Reaction Pathway Differentiation Enabled Fingerprinting Signal for Single Nucleotide Variant Detection
Source: Adv Sci (Weinh). 2025 Feb 4;12(12):2412680. doi: 10.1002/advs.202412680 (PMC11948007; doi:10.1002/advs.202412680)
Supplement: Supplementary file 1 — Supporting Information [file ADVS-12-2412680-s001.pdf]

ADVANCED SCIENCE
Open Access

Supporting Information

for *Adv. Sci.*, DOI 10.1002/advs.202412680

Reaction Pathway Differentiation Enabled Fingerprinting Signal for Single Nucleotide Variant Detection

Huixiao Yang, Linghao Zhang, Xinmiao Kang, Yunpei Si, Ping Song and Xin Su**

Supporting Information

Reaction Pathway Differentiation Enabled Fingerprinting Signal for Single Nucleotide Variant Detection

Huixiao Yang,[1] Linghao Zhang, Xinmiao Kang, Yunpei, Si, Ping Song and Xin Su**

* Corresponding authors.

This PDF file includes:

Supporting Notes for kinetics simulation

Supporting Figures: Figure S1 to Figure S25

Supporting Table: Table S1 to Table S6

Supporting Note

Kinetic fitting and simulation of the probe.

The reaction equations involving SNV in differential reaction pathway probe (DRPP):

(1) SNV + S-Probe $\underset{k_2}{\overset{k_1}{\rightleftharpoons}}$ Messenger DNA + SNV-S-Probe complex

(2) Messenger DNA + P-Probe $\xrightarrow{k_5}$ Messenger DNA-P-Probe complex

(3) Messenger DNA-P-Probe complex + λ Exo $\underset{k_7}{\overset{k_6}{\rightleftharpoons}}$ λ Exo complex

(4) λ Exo complex $\xrightarrow{k_8}$ Opened P-Probe + dNMP +λ Exo

(5) Opened P-Probe $\xrightarrow{k_9}$ P-Probe

The reaction equations involving WT in differential reaction pathway probe (DRPP):

(1) WT + S-Probe $\underset{k_4}{\overset{k_3}{\rightleftharpoons}}$ Messenger DNA + WT-S-Probe complex

(2) Messenger DNA + P-Probe $\xrightarrow{k_5}$ Messenger DNA-P-Probe complex

(3) Messenger DNA-P-Probe complex + λ Exo $\underset{k_7}{\overset{k_6}{\rightleftharpoons}}$ λ Exo complex

(4) λ Exo complex $\xrightarrow{k_8}$ Opened P-Probe + dNMP +λ Exo

(5) Opened P-Probe + WT $\xrightarrow{k_{10}}$ WT-P-Probe complex

The reaction equations in shared reaction pathway probe (SRPP):

(1) SNV + TMSD-Probe $\xrightarrow{k_1}$ SNV-reporter + SNV-TMSD-Probe complex

(2) WT + TMSD-Probe $\xrightarrow{k_3}$ WT-reporter + WT-TMSD-Probe complex

The differential equations were established based on the following assumptions:

(1) the TMSD reaction is irreversible if there is no reverse toehold;

(2) enzyme kinetics follows a typical Michaelis–Menten kinetics model;

(3) the TMSD reaction and enzymatic reaction are independent, and their kinetics do not affect

each other;

(4) the refold and hybridization process is irreversible;

(5) the digestion of messenger DNA by λ Exo, generating dNMP, does not influence the entire reaction process;

(6) During the SRPP reaction, the variation in SNV/WT-reporter concentrations

The differential equations involving SNV in DRPP:

(1) d[SNV]/d[t]=-k_1 [SNV][S-Probe]+k_2 [Messenger DNA][SNV-S-Probe complex];

(2) d[S-Probe]/d[t]=-k_1 [SNV][S-Probe]+k_2 [Messenger DNA][SNV-S-Probe complex];

(3) d[Messenger DNA]/d[t]=k_1 [SNV][S-Probe]-k_2 [Messenger DNA][SNV-S-Probe complex] -k_5 [Messenger DNA][P-Probe];

(4) d[SNV-S-Probe complex]/d[t]=k_1 [SNV][S-Probe]-k_2 [Messenger DNA][SNV-S-Probe complex];

(5) d[P-Probe]/d[t]=-k_5 [Messenger DNA][P-Probe]+k_9[opened P-Probe];

(6) d[Messenger DNA-P-Probe complex]/d[t]=k_5[Messenger DNA][P-Probe]-k_6[Messenger DNA-P-Probe complex][λ Exo]+k_7[λ Exo complex];

(7) d[λ Exo complex]/d[t]= k_6[Messenger DNA-P-Probe complex][λ Exo]-k_7[λ Exo complex]-k_8[opened P-Probe][dNMP][λ Exo] ;

(8) d[opened P-Probe]/d[t]=-k_9[opened P-Probe] ;

The differential equations involving WT in DRPP:

(1) d[S-Probe]/d[t]= -k_3 [WT][S-Probe]+k_4 [Messenger DNA][WT-S-Probe complex];

(2) d[Messenger DNA]/d[t]= k_3 [WT][S-Probe]-k_4 [Messenger DNA][WT-S-Probe complex]-k_5 [Messenger DNA][P-Probe];

(3) d[WT]/d[t]=-k_3 [WT][S-Probe]+k_4 [Messenger DNA][WT-S-Probe complex]-k_{10}[WT][opened Probe];

(4) d[WT-S-Probe complex]/d[t]=k_3 [WT][S-Probe]-k_4 [Messenger DNA][WT-S-Probe complex];

(5) d[P-Probe]/d[t]=-k_5 [Messenger DNA][P-Probe];

(6) d[Messenger DNA-P-Probe complex]/d[t]=k_5[Messenger DNA][P-Probe]-k_6[Messenger DNA-P-Probe complex][λ Exo]+k_7[λ Exo complex];

(7) d[λ Exo complex]/d[t]= k_6[Messenger DNA-P-Probe complex][λ Exo]-k_7[λ Exo complex]-k_8[opened P-Probe][dNMP][λ Exo];

(8) d[opened P-Probe]/d[t]= -k_{10}[opened P-Probe][WT];

(9) d[WT-P-Probe complex]/d[t]= k_{10}[opened P-Probe][WT];

The differential equations in SRPP:

(1) d[SNV]/d[t]=-k_1 [SNV][TMSD-Probe];

(2) d[TMSD-Probe]/d[t]=-k_1 [SNV][TMSD-Probe];

(3) d[SNV-reporter]/d[t]=k_1 [SNV][TMSD-Probe];

(4) d[SNV-TMSD-Probe complex]/d[t]=k_1 [SNV][TMSD-Probe];

(5) d[WT]/d[t]=-k_3 [WT][TMSD-Probe];

(6) d[TMSD-Probe]/D[t]=-k_3 [WT][TMSD-Probe];

(7) d[WT-reporter]/D[t]=k_3 [WT][TMSD-Probe];

(8) d[WT-TMSD-Probe complex]/d[t]=k_3 [WT][TMSD-Probe];

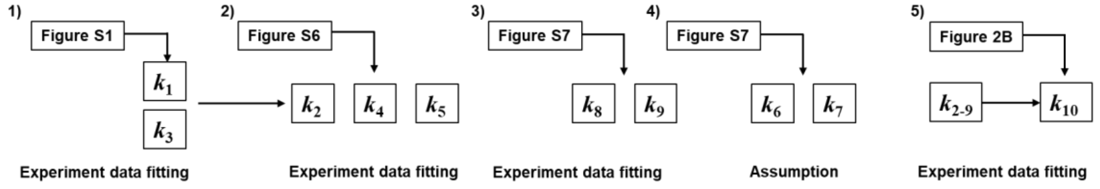

For kinetic simulations, some rate constants were obtained by fitting experiment data. To fit the fluorescence kinetics, the fluorescently labeled DNA species such as opened P-Probe and SNV/WT-reporter at a certain time was determined by (F_t-$F_{control}$)/(F_{target}-$F_{control}$)×[target], where F_t represents real-time fluorescence, $F_{control}$ represents the background signal, [target] represents the determined concentration, and F_{target} represents the fluorescence intensity of [target]. As shown in Figure S1, we first fitted the strand replacement rate of SRPP. TMSD probe features a forward toehold length of 7 nt, and lacks a reverse Toehold, implying irreversible strand displacement. Through the kinetic equation of SRPP and the experimental data in **Figure S1A**, derived fitted values for k_1 and k_3 as 5.5×10^4 $M^{-1}s^{-1}$ and 8.1×10^2 $M^{-1}s^{-1}$. Subsequently, we analyze Figure S6 to determine the rate constants governing the strand displacement of messenger with P-Probe in DRPP. As this process involves a cascade reaction, it is imperative to fit the rate constants k_2 and k_4 in conjunction with the preceding determinations of k_1 and k_3. The final rate constants k_2, k_4, and k_5 are 1.2×10^3 $M^{-1}s^{-1}$, 1.7×10^3 $M^{-1}s^{-1}$, 5.1×10^4 $M^{-1}s^{-1}$. The concentration of λ Exo was converted to apparent molar concentration, where 50 U/mL corresponds to 5.04 nM [1]. Constants of enzyme catalysis, k_m, and k_8 were determined based on enzyme kinetics results from **Figure S7**: k_m was found to be 320 nM, and k_8 was 4.5 s^{-1}. Constants of refold, k_9 was determined as 1.5×10^5 $M^{-1}s^{-1}$ by fitting the data in **Figure S7**. The other rate constants were obtained from assumption. Assuming at least 95% enzyme-substrate binding in enzyme-saturated conditions when the substrate is in excess, we utilized the reaction (3) to derive k_6/k_7 = 95%/5% × [Messenger DNA P-Probe complex]. Taking 1000 nM as the concentration of messenger DNA P-Probe complex, the Michaelis-Menten equation led to the determination of k_6 and k_7 as 1.35×10^6 $M^{-1}s^{-1}$ and 0.68 s-

^{1}, respectively.

Based on the rate constants k_2-k_9 already obtained above and the experimental data in **Figure 2B**, we conclude that the rate constant k_{10} for WT hybridization with open-P-Probe is 2.5×10^5 $M^{-1}s^{-1}$. The reaction rate constants are summarized in following table.

SRPP	$k_1 = 5.5\times10^4\ M^{-1}s^{-1}$
	$k_3 = 8.1\times10^2\ M^{-1}s^{-1}$
DRPP	$k_2 = 1.2\times10^3\ M^{-1}s^{-1}$
	$k_4 = 1.7\times10^3\ M^{-1}s^{-1}$
	$k_5 = 5.1\times10^4\ M^{-1}s^{-1}$
	$k_6 = 2.7\times10^3\ M^{-1}s^{-1}$
	$k_7 = 0.68\ s^{-1}$
	$k_8 = 4.5\ s^{-1}$
	$k_9 = 1.5\times10^5\ M^{-1}s^{-1}$
	$k_{10} = 2.5\times10^5\ M^{-1}s^{-1}$

Supporting Figure

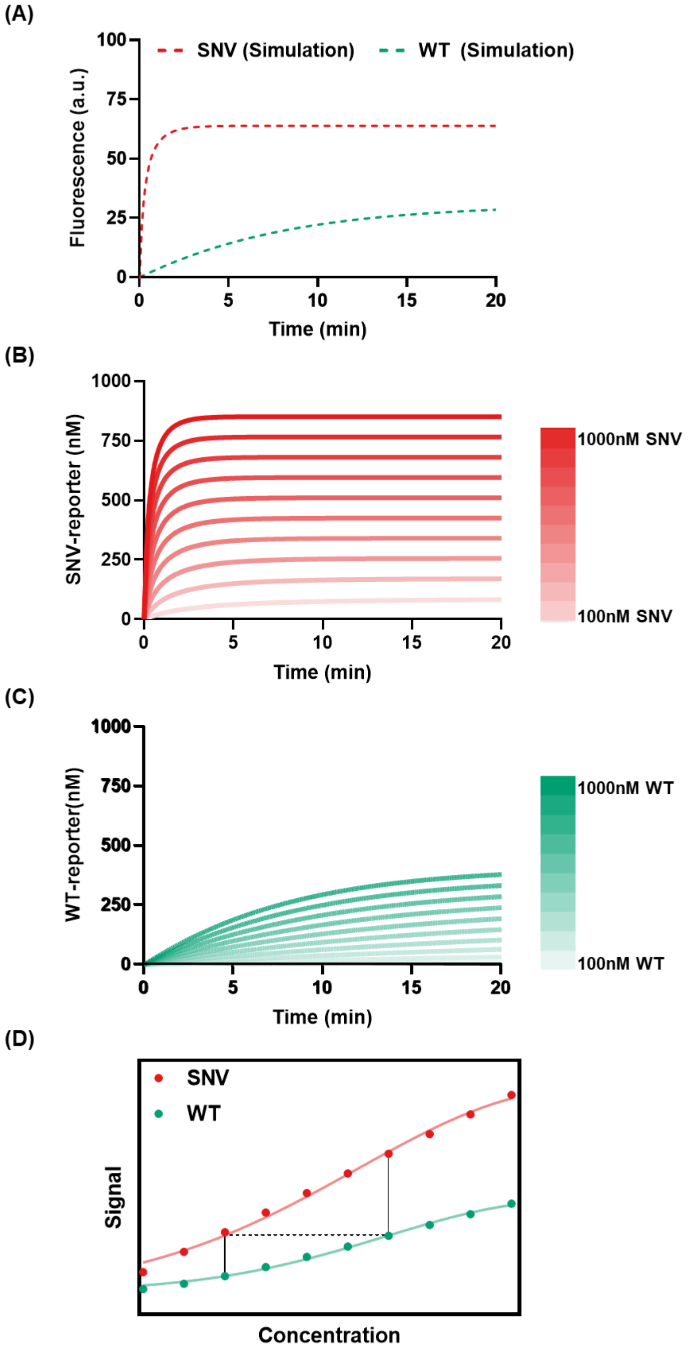

Figure S1. Kinetics simulation of SRPP with SNV/WT reactions (as exemplified by the TMSD probe). (A) SRPP kinetic simulations of SNV and WT reactions. (B) Kinetics simulation of SRPP reactions at different SNV concentrations. (C) Kinetics simulation of SRPP reaction at different WT concentrations. (D) TMSD probe signal of the reaction with different concentrations of SNV and WT after 20 minutes. The simulation process is detailed in Supplementary Note, and the sequences are detailed in Table S4.

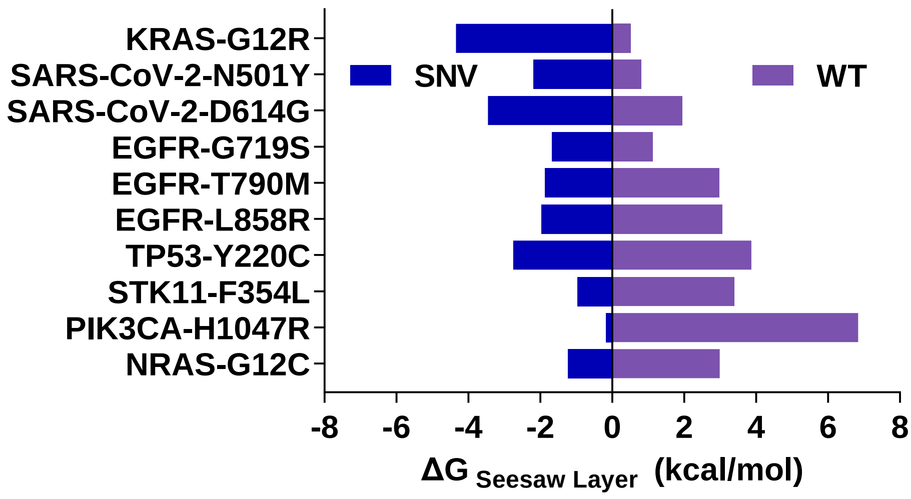

Figure S2. Free energy of the strand displacement reaction in Seesaw Layer for the 10 mutant genes (sequence details are given in Table S5), and the free energy change in the Seesaw layer was calculated by the MEF function in NUPACK[2], $\Delta G_{\text{Seesaw Layer}} = \Delta G_{\text{After the reaction}} - \Delta G_{\text{Before the reaction}}$. Taking *KRAS-G12R* as an example, the energy change of SNV after the reaction with the Seesaw layer, $\Delta G_{\text{After the reaction}}$ = -67.84 kcal/mol, $\Delta G_{\text{Before the reaction}}$ = -63.5 kcal/mol, thus, SNV's $\Delta G_{\text{Seesaw Layer}}$ = -4.34 kcal/mol; the energy change after the reaction of WT with the seesaw layer, $\Delta G_{\text{After the reaction}}$ = -64.04 kcal/mol, $\Delta G_{\text{Before the reaction}}$ = -64.56 kcal/mol, thus, WT's $\Delta G_{\text{Seesaw Layer}}$ = 0.52 kcal/mol.

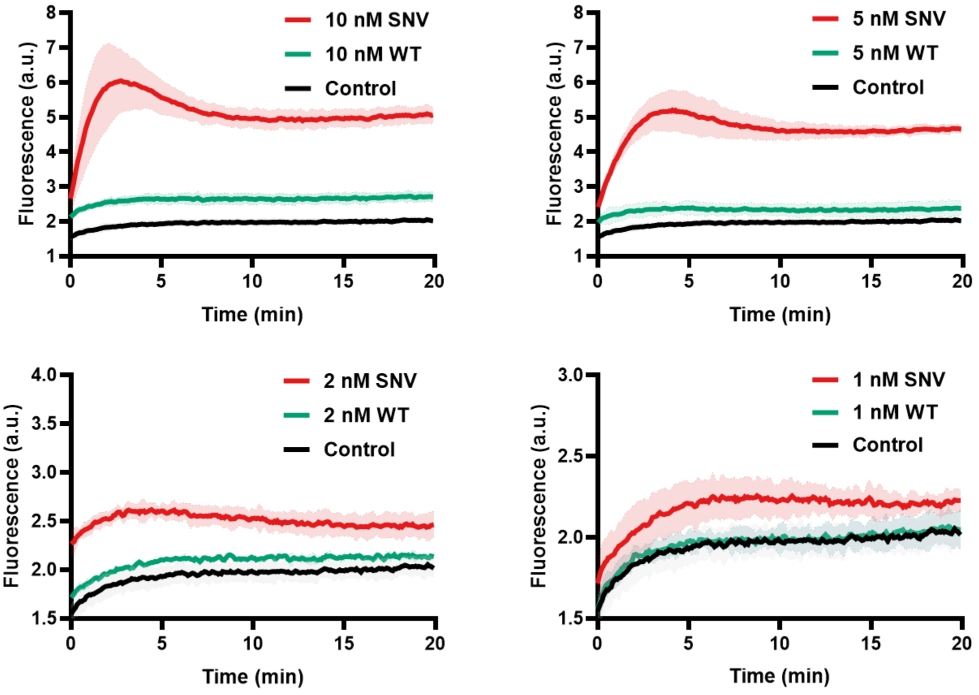

Figure S3. Time-course fluorescence of SNV and WT of 1-10 nM detected by DRPP. The forward toehold and reverse toehold of S-Probe were 7 and 5 nt, respectively. The mismatch site was located at the 7 nt of the 3' end of the forward toehold of S-Probe. All nucleic acid species in DRPP were 100 nM, and λ Exo was 50 U/mL. All reactions were performed at 37 °C in 1×DRPP buffer. Data are mean±S.D. (n = 3 independent experiments).

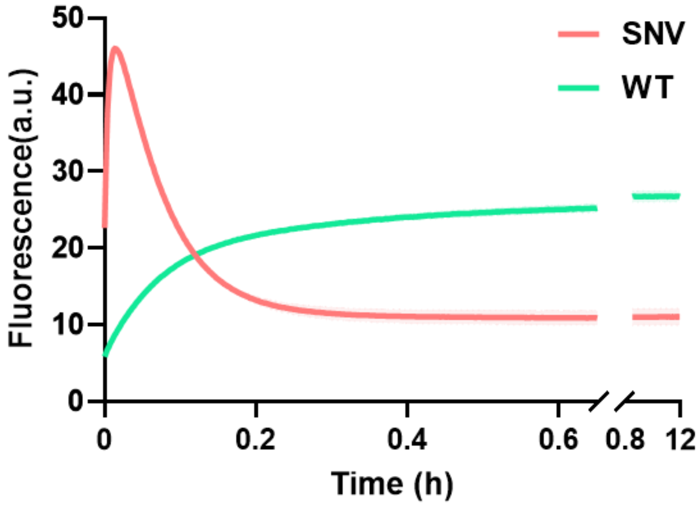

Figure S4. Time-course fluorescence of SNV and WT over 12 hours. The mismatch site was located at the 7 nt of the 3' end of the forward toehold of S-Probe. All nucleic acid species in

DRPP were 100 nM, and λ Exo was 50 U/mL. All reactions were performed at 37 °C in 1×DRPP buffer. Data are mean±S.D. (n = 3 independent experiments).

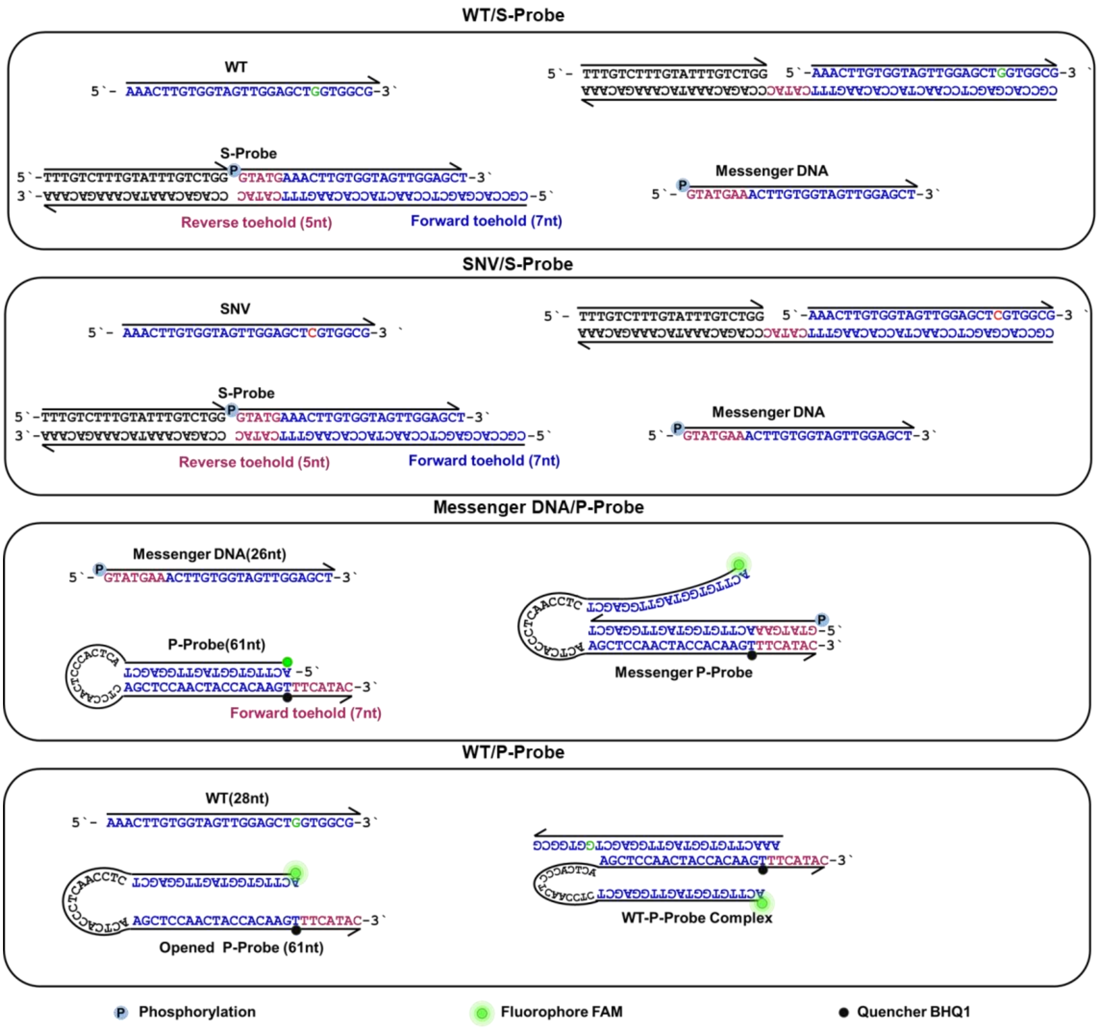

Figure S5. Details of hybridization regions SNV/WT with the nucleic acid species in DRPP.

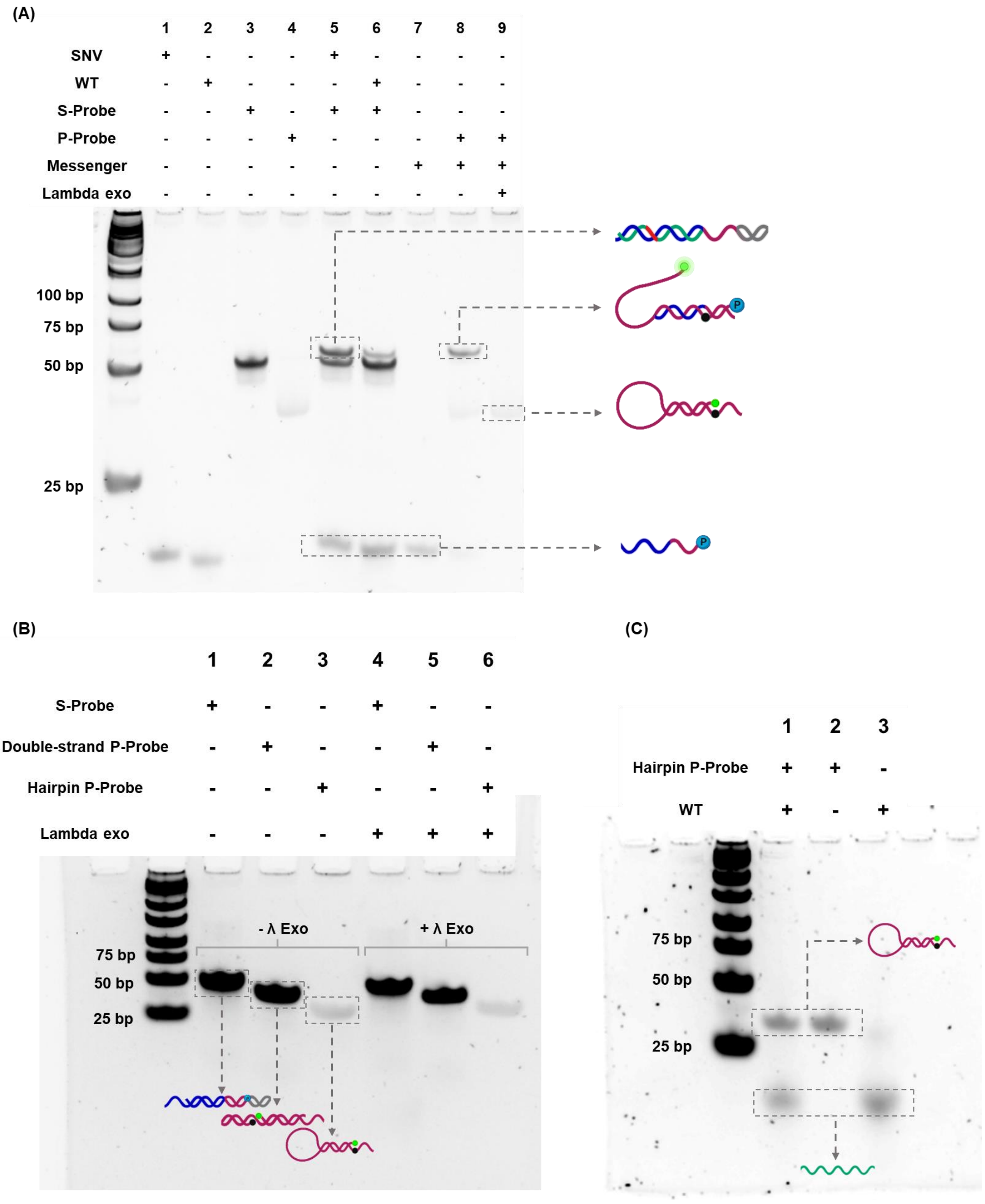

Figure S6. (A) Polyacrylamide gel (15%) electrophoresis characterization of the reaction product of SNV/WT with DRPP. Lanes 5 and 6 represent the producut of the strand displacement reaction of SNV and WT with S-Probe, and lane 8 represents the strand displacement reaction of messenger DNA and S-Probe to form the messenger DNA-P-Probe complex. Lane 9 illustrates the digestion of the messenger DNA-P-Probe complex by λ Exo, followed by subsequent refolding to form P-Probe. (B) Polyacrylamide gel (15%) electrophoresis characterized the interaction of λ Exo on the structure of the S-probe, double-

stranded P-probe, and hairpin P-probe. (C) Polyacrylamide gel (15%) electrophoresis characterized the interaction of the hairpin P-Probe and WT reactions.

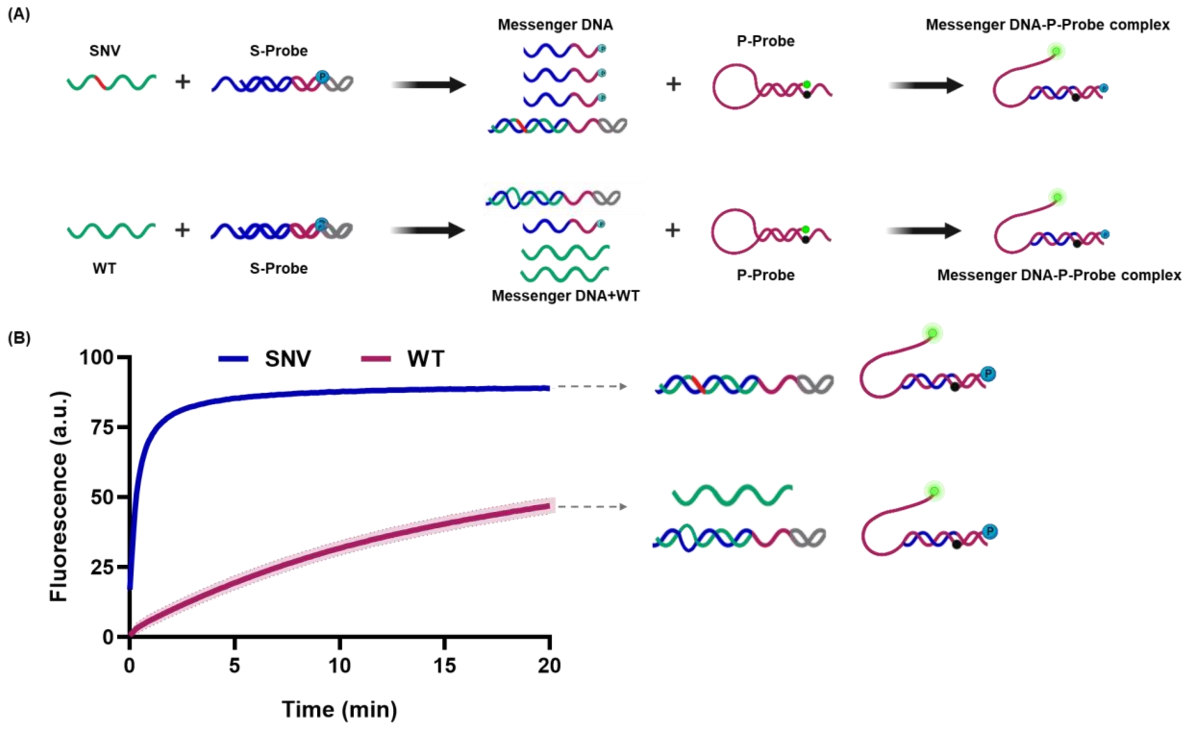

Figure S7. (A) Schematic of the two-strand displacement reactions in P-Probe. (B) Fluorescence kinetics of the reaction in panel A to obtain DRPP kinetic simulation parameters (see Supplementary note 1 for details). The mismatch site was located at the 7 nt of the 3' end of the forward toehold of S-Probe. All reactions were performed at 37 °C in 1×DRPP buffer. All nucleic acid species were 1000 nM. Data are mean±S.D. (n = 3 independent experiments).

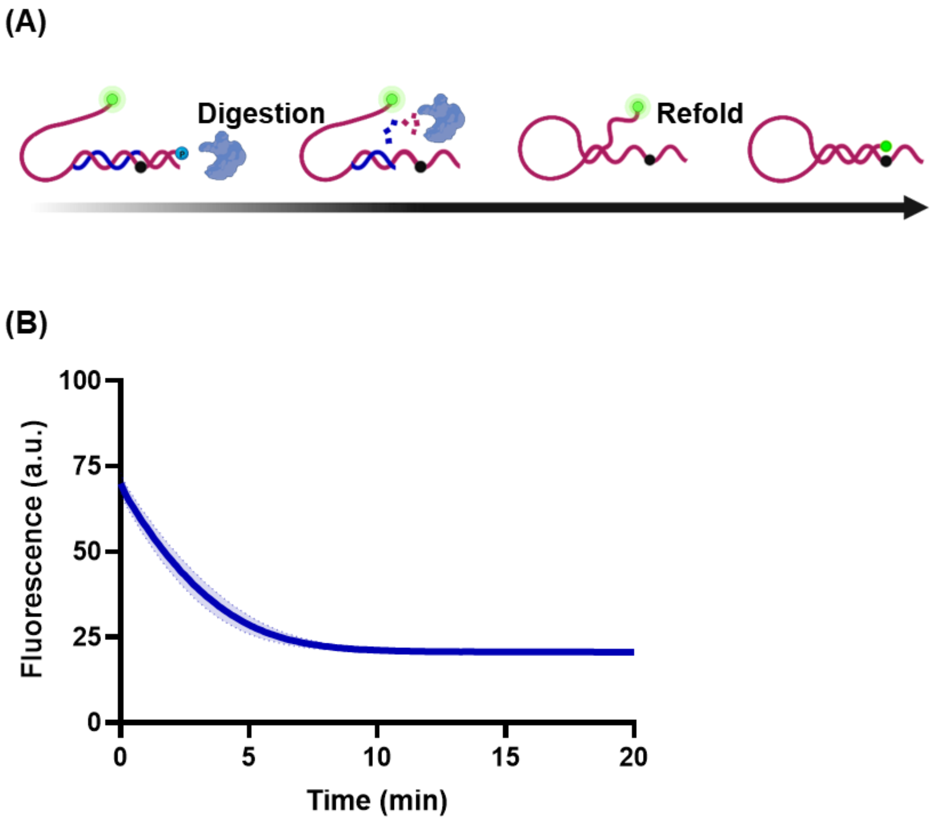

Figure S8. (A) Schematic of λ Exo digestion of messenger DNA-P-Probe complex and P-Probe re-fold process. (B) Fluorescence kinetics of the reaction in panel A to obtain DRPP kinetic simulation parameters (see **Supplementary note** 1 for details). All reactions were performed at 37 °C in 1×DRPP buffer. All nucleic acid species were 1000 nM, and λ Exo was 50 U/mL. Data are mean±S.D. (n = 3 independent experiments).

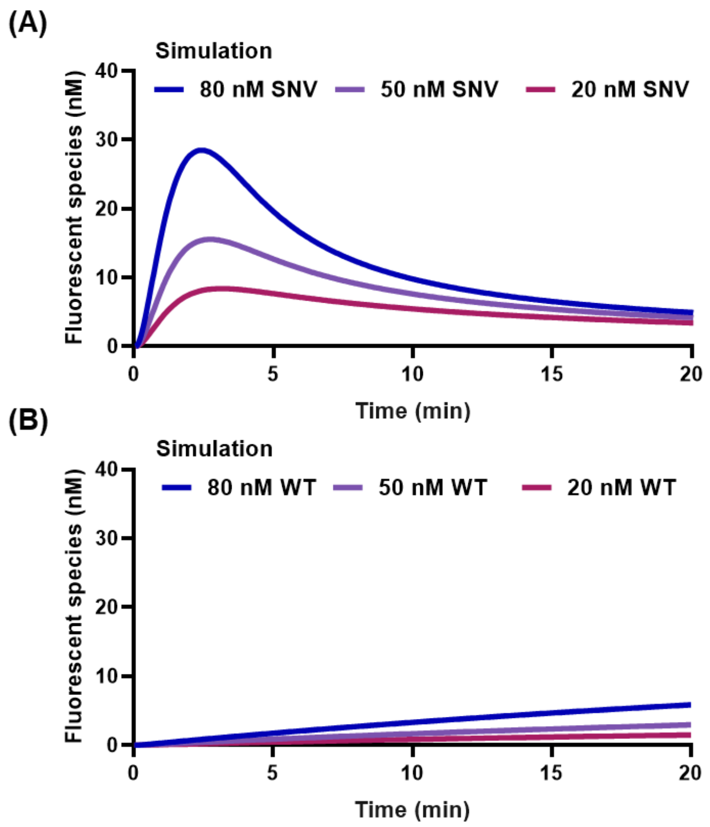

Figure S9. (A) Simulated kinetics of SNV reactions with DRPP. (B) Simulated kinetics of WT reactions with DRPP. (See **Supporting Information note** for parameter setting). Detailed information regarding the hybridization regions of the SNV/WT, P-Probe, and messenger DNA is shown in **Figure S4.**

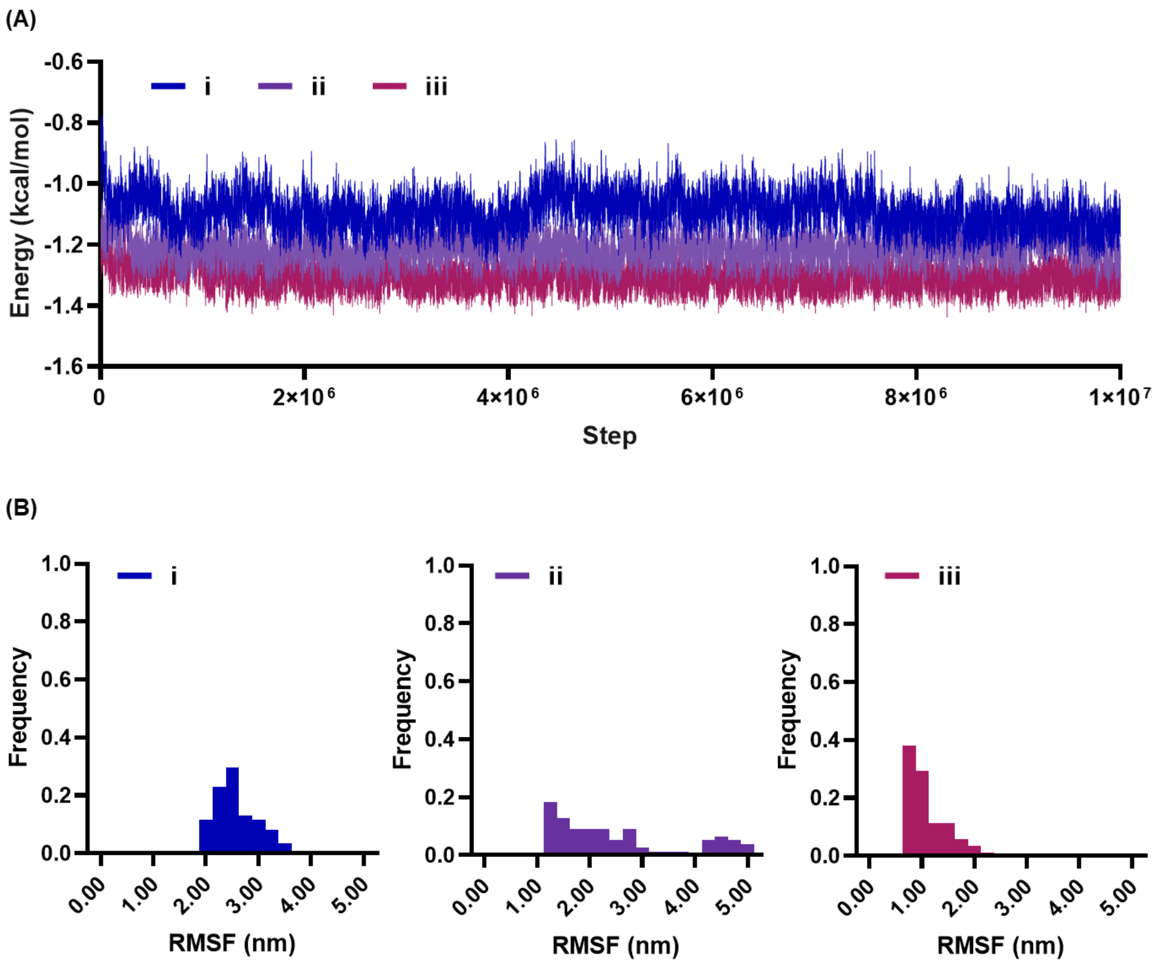

Figure S10. Molecular dynamic simulation based on the coarse-grained model of the WT and opened P-Probe reaction. (A) Energy landscapes for states i, ii, and iii in **Figure 3C**. (B) Root mean square fluctuation (RMSF) of states i, ii, and iii in **Figure 3C**. oxView was used to build i、ii、iii structures. The position and orientation of each base can be adjusted, providing a more intuitive graphical representation of DNA nanostructures.

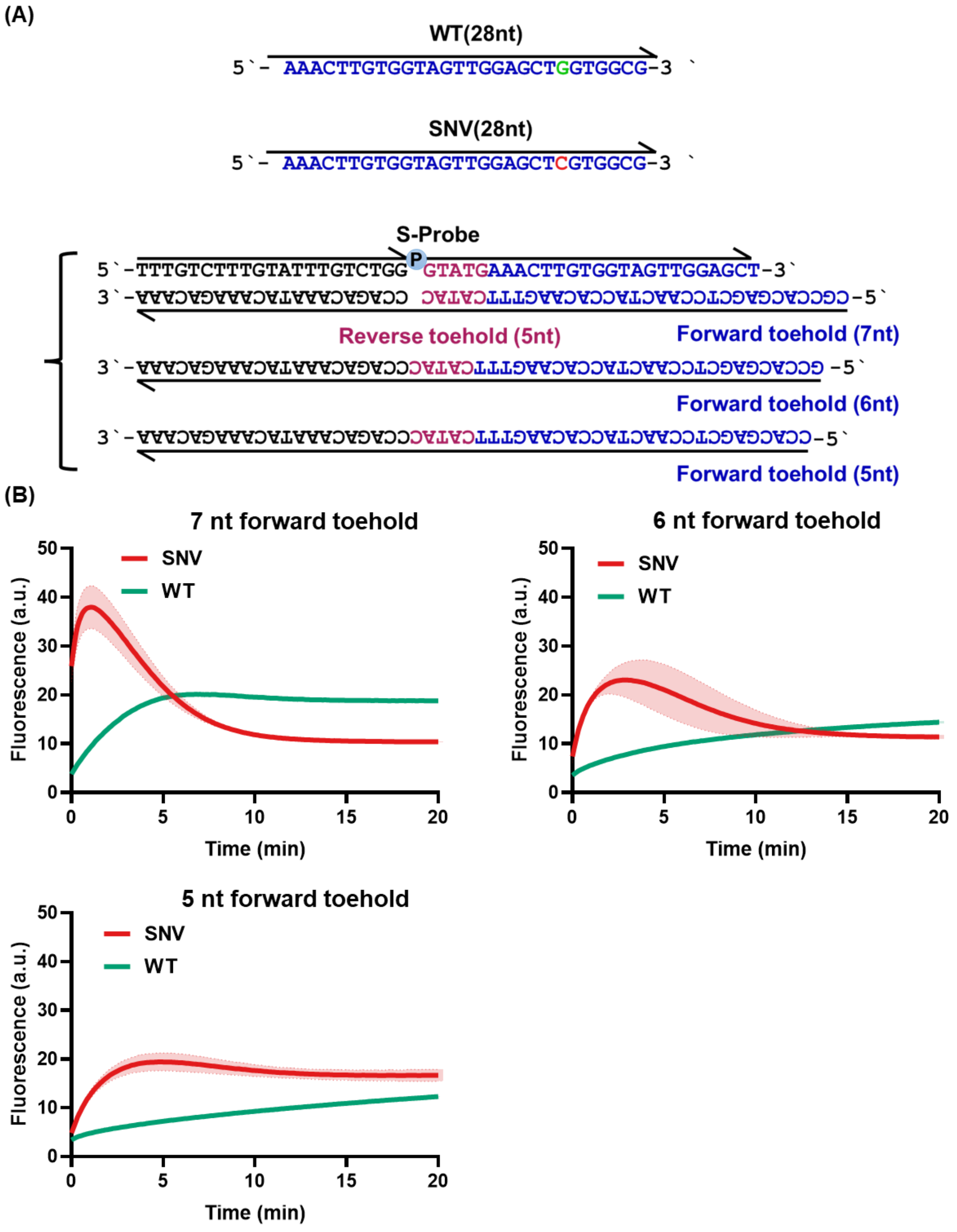

Figure S11. Exploration of the effect of S-Probe forward toehold length on DRPP (hairpin P-Probe) performance. (A) The forward toehold length of the S-Probe varied from 5 to 7 nt, while the reverse toehold length was fixed at 5 nt. (B) Fluorescence signals of SNV/WT by different S-Probe. The mismatch site was located at the 7 nt of the 3' end of the forward toehold of S-Probe. All reactions were performed at 37 °C in 1×DRPP buffer. All targets and nucleic acid species in DRPP were 100 nM, and λ Exo was 50 U/mL. Data are mean±S.D. (n = 3 independent experiments).

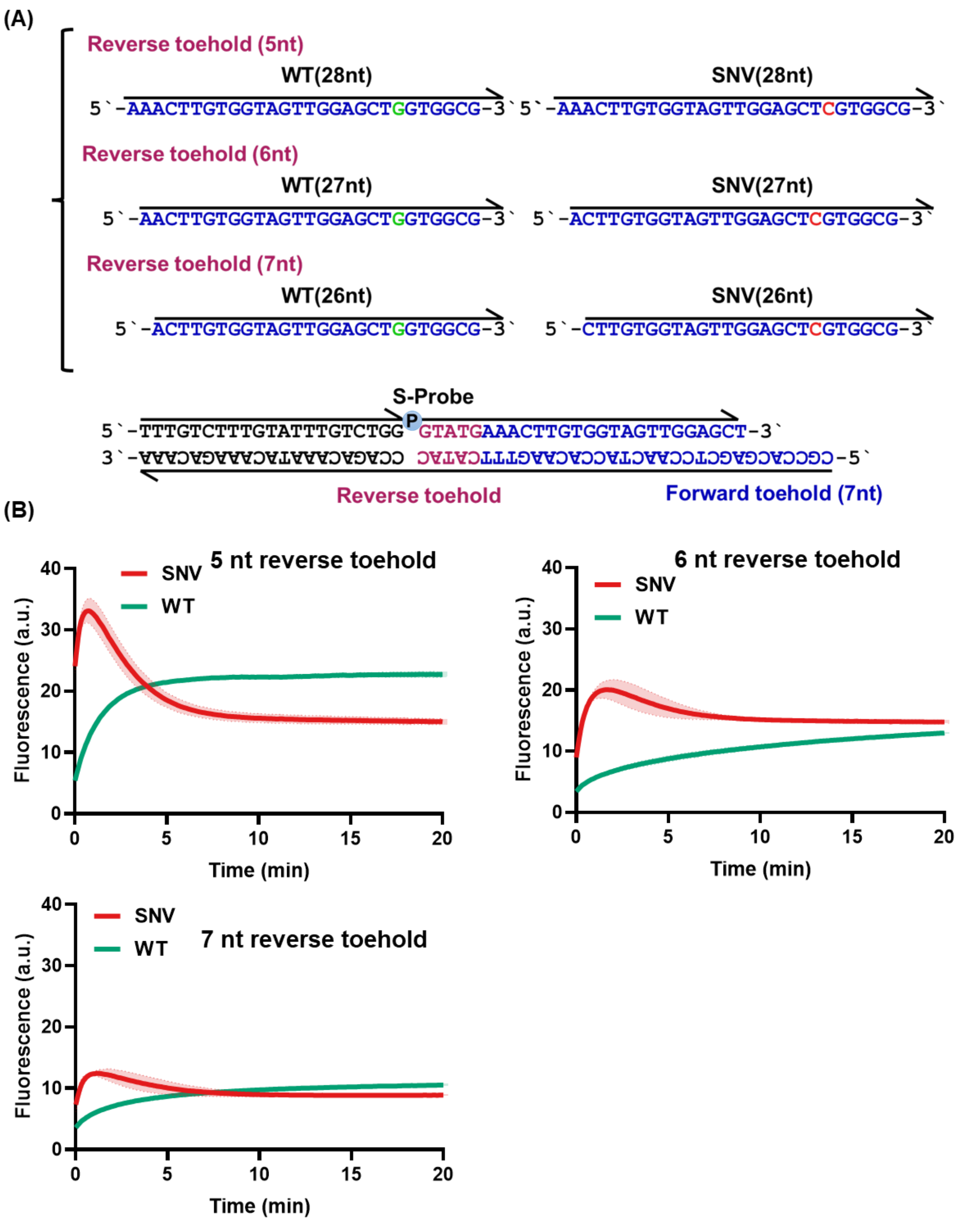

Figure S12. Exploration of the effect of S-Probe reverse toehold length on DRPP (hairpin P-Probe) performance. (A) The reverse toehold length of the S-Probe varied from 5 to 7 nt, while the forward toehold length was fixed at 7 nt. (B) Fluorescence signals of SNV/WT by different S-Probe. The mismatch site was located at the 7 nt of the 3' end of the forward toehold of S-Probe. All reactions were performed at 37 °C in 1×DRPP buffer. All targets and nucleic acid species in DRPP were 100 nM, and λ Exo was 50 U/mL. Data are mean±S.D. (n = 3 independent experiments).

(A)

Substitutions at different positions

Target	Detection region
Match	AAACTTGTGGTAGTTGGAGCTCGTGGCG
Mismatch 1(01G->C)	AAACTTGTGGTAGTTGGAGCTCGTGGCC
Mismatch 2(02C->G)	AAACTTGTGGTAGTTGGAGCTCGTGGGG
Mismatch 3(03G->C)	AAACTTGTGGTAGTTGGAGCTCGTGCCG
Mismatch 4(04G->C)	AAACTTGTGGTAGTTGGAGCTCGTCGCG
Mismatch 5(05T->C)	AAACTTGTGGTAGTTGGAGCTCGCGGCG
Mismatch 6(06G->C)	AAACTTGTGGTAGTTGGAGCTCCTGGCG
Mismatch 7(07C->G)	AAACTTGTGGTAGTTGGAGCTGGTGGCG
Mismatch 8(08T->C)	AAACTTGTGGTAGTTGGAGCCCGTGGCG
Mismatch 9(09C->G)	AAACTTGTGGTAGTTGGAGGTCGTGGCG
Mismatch 10(10G->C)	AAACTTGTGGTAGTTGGACCTCGTGGCG
Mismatch 11(11A->C)	AAACTTGTGGTAGTTGGCGCTCGTGGCG
Mismatch 12(12G->C)	AAACTTGTGGTAGTTGCAGCTCGTGGCG
Mismatch 13(13G->C)	AAACTTGTGGTAGTTCGAGCTCGTGGCG
Mismatch 14(14T->C)	AAACTTGTGGTAGTCGGAGCTCGTGGCG
Mismatch 15(15T->C)	AAACTTGTGGTAGCTGGAGCTCGTGGCG
Mismatch 16(16G->C)	AAACTTGTGGTACTTGGAGCTCGTGGCG
Mismatch 17(17A->C)	AAACTTGTGGTCGTTGGAGCTCGTGGCG
Mismatch 18(18T->C)	AAACTTGTGGCAGTTGGAGCTCGTGGCG
Mismatch 19(19G->C)	AAACTTGTGCTAGTTGGAGCTCGTGGCG
Mismatch 20(20G->C)	AAACTTGTCGTAGTTGGAGCTCGTGGCG
Mismatch 21(21T->C)	AAACTTGCGGTAGTTGGAGCTCGTGGCG
Mismatch 22(22G->C)	AAACTTCTGGTAGTTGGAGCTCGTGGCG
Mismatch 23(23T->C)	AAACTCGTGGTAGTTGGAGCTCGTGGCG
Mismatch 24(24T->G)	AAACGTGTGGTAGTTGGAGCTCGTGGCG
Mismatch 25(25C->G)	AAAGTTGTGGTAGTTGGAGCTCGTGGCG
Mismatch 26(26A->G)	AAGCTTGTGGTAGTTGGAGCTCGTGGCG
Mismatch 27(27A->G)	AGACTTGTGGTAGTTGGAGCTCGTGGCG
Mismatch 28(27A->G)	GAACTTGTGGTAGTTGGAGCTCGTGGCG

(B)

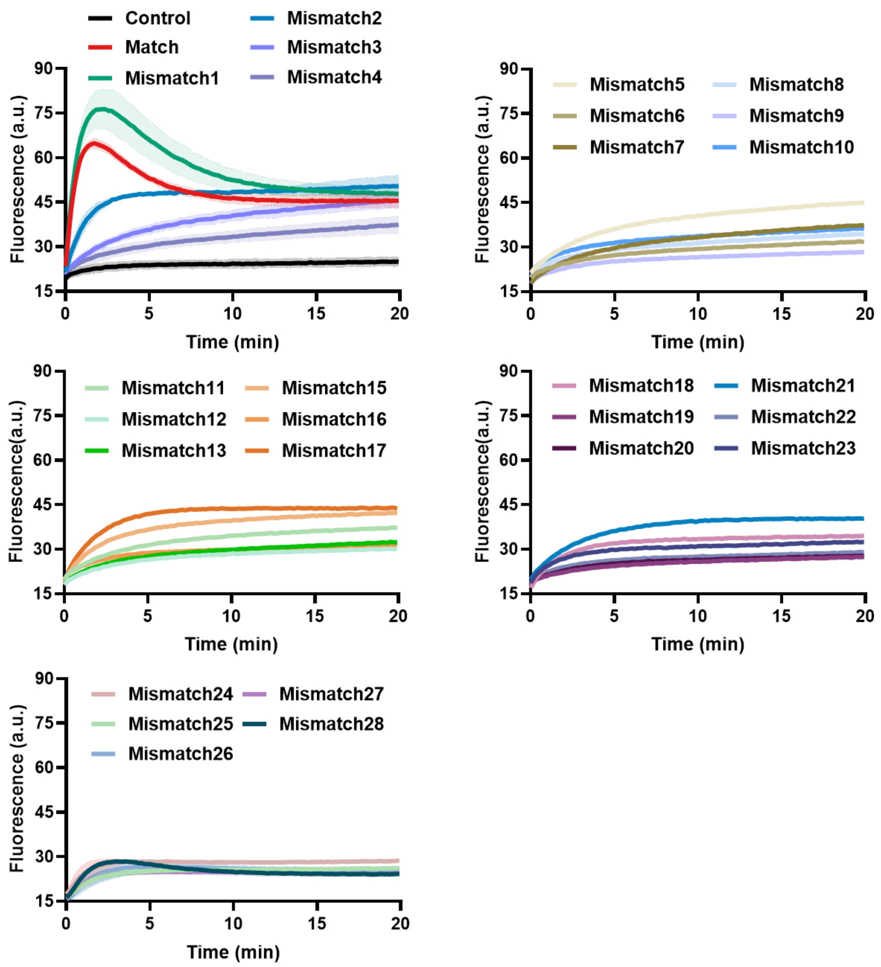

Figure S13. Exploring the effect of mismatch site of target and S-Probe on the DRPP (hairpin P-Probe) performance. (A) Sequences of the match and mismatch targets. The mismatch targets for single-nucleotide mismatch with S-Probe at different regions (toehold region or branch migration region). (B) Fluorescence signals the targets in panel A. The forward toehold and reverse toehold of S-Probe were 7 and 5 nt, respectively. All reactions were performed at 37 °C in 1×DRPP buffer. All targets and nucleic acid species in DRPP were 100 nM, and λ Exo was 50 U/mL. Data are mean±S.D. (n = 3 independent experiments).

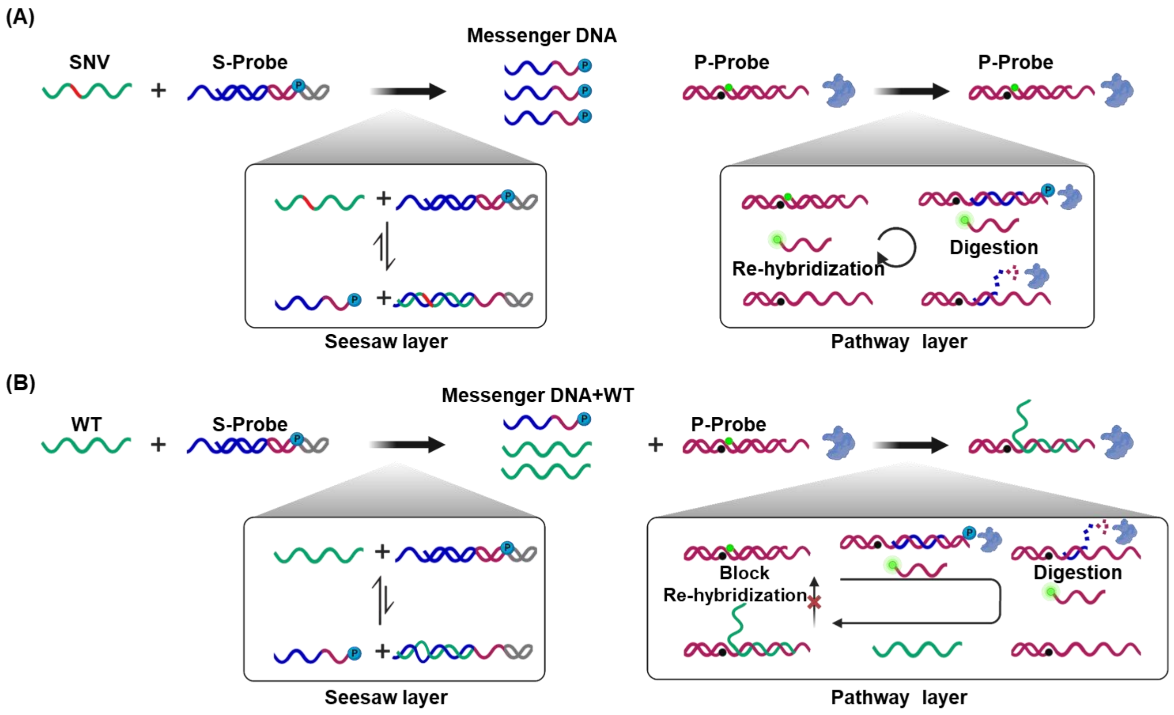

Figure S14. Schematic representation of the scenarios of DRPP which uses double-stranded P-Probe. All other conditions are the same as those of hairpin P-Probe.

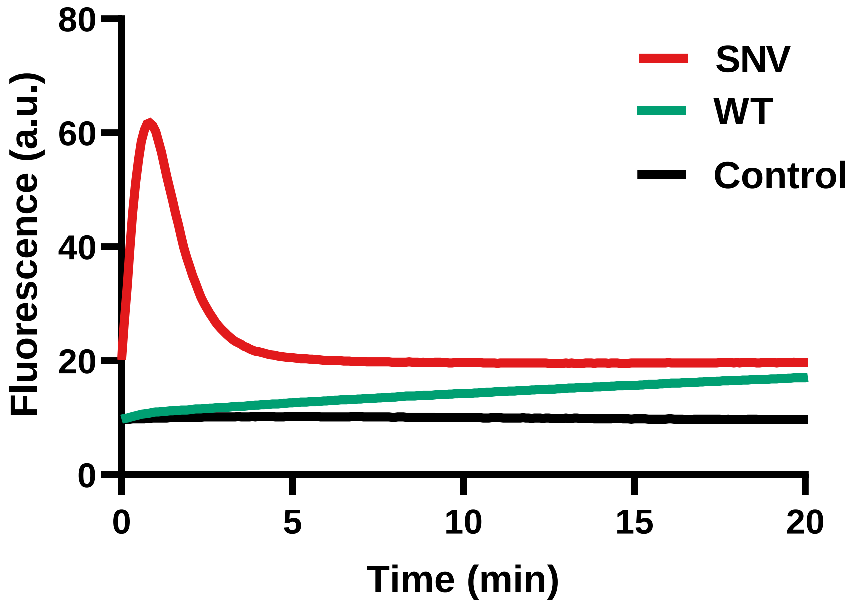

Figure S15. Fluorescence signals of SNV/WT detected by DRPP (double-stranded P-Probe). The forward toehold and reverse toehold of S-Probe were 7 and 5 nt, respectively. The mismatch site was located at the 7 nt of the 3' end of the forward toehold of S-Probe. All reactions were performed at 37 °C in 1×DRPP buffer. All targets and nucleic acid species in DRPP were 100 nM, and λ Exo was 50 U/mL.

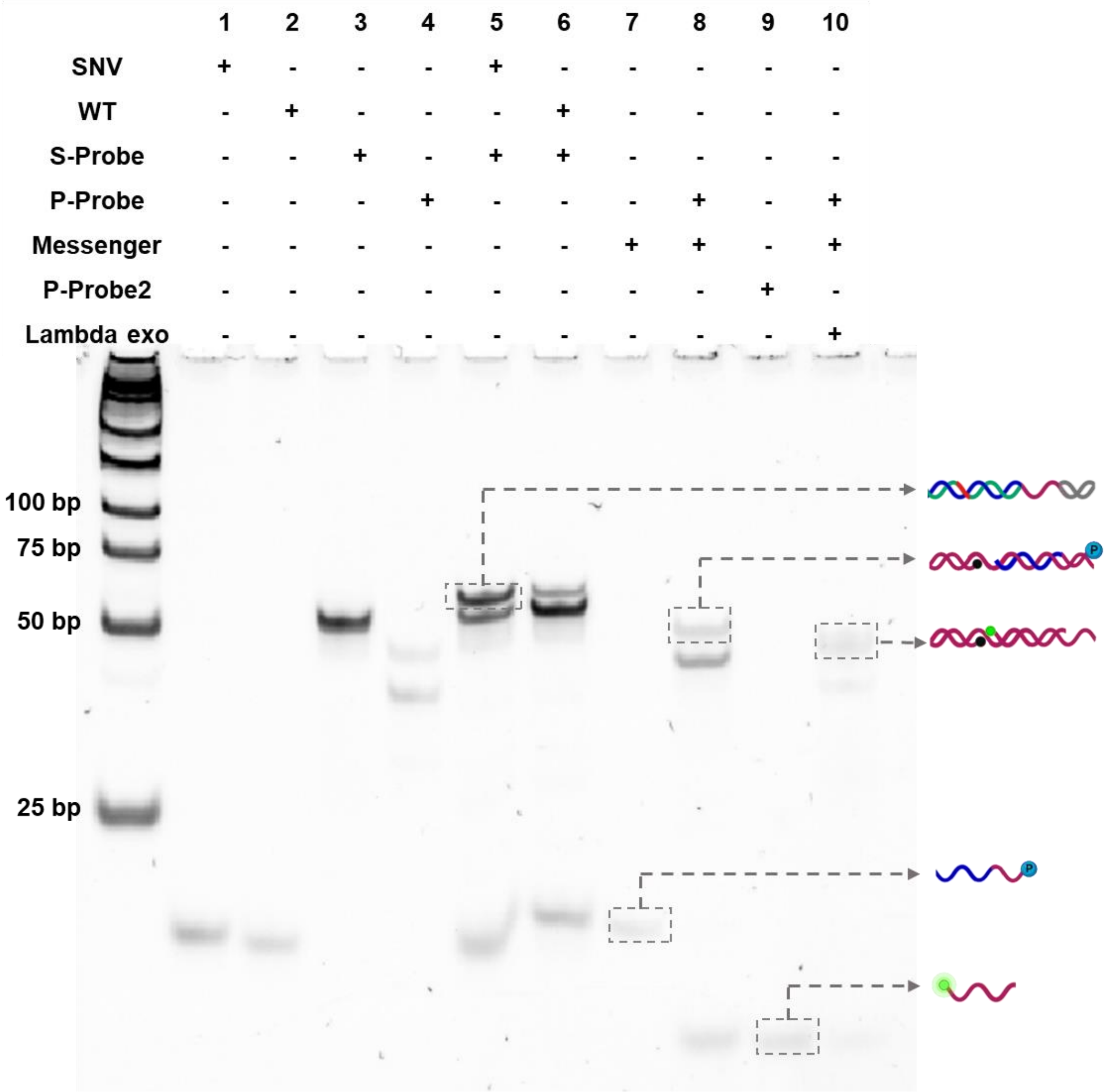

Figure S16. Polyacrylamide gel (15%) electrophoresis characterization of the reaction product of SNV/WT with DRPP (double-stranded P-Probe). Lanes 5 and 6 represent the strand displacement product of SNV and WT with S-Probe, and lane 9 represents the strand displacement product of messenger DNA with P-Probe to form the messenger DNA-P-Probe complex, which releases fluorophore strands. Lane 10 indicates that the λ Exo digests the messenger DNA-P-Probe complex and subsequently fluorophore strands rehybridizes to form the P-Probe.

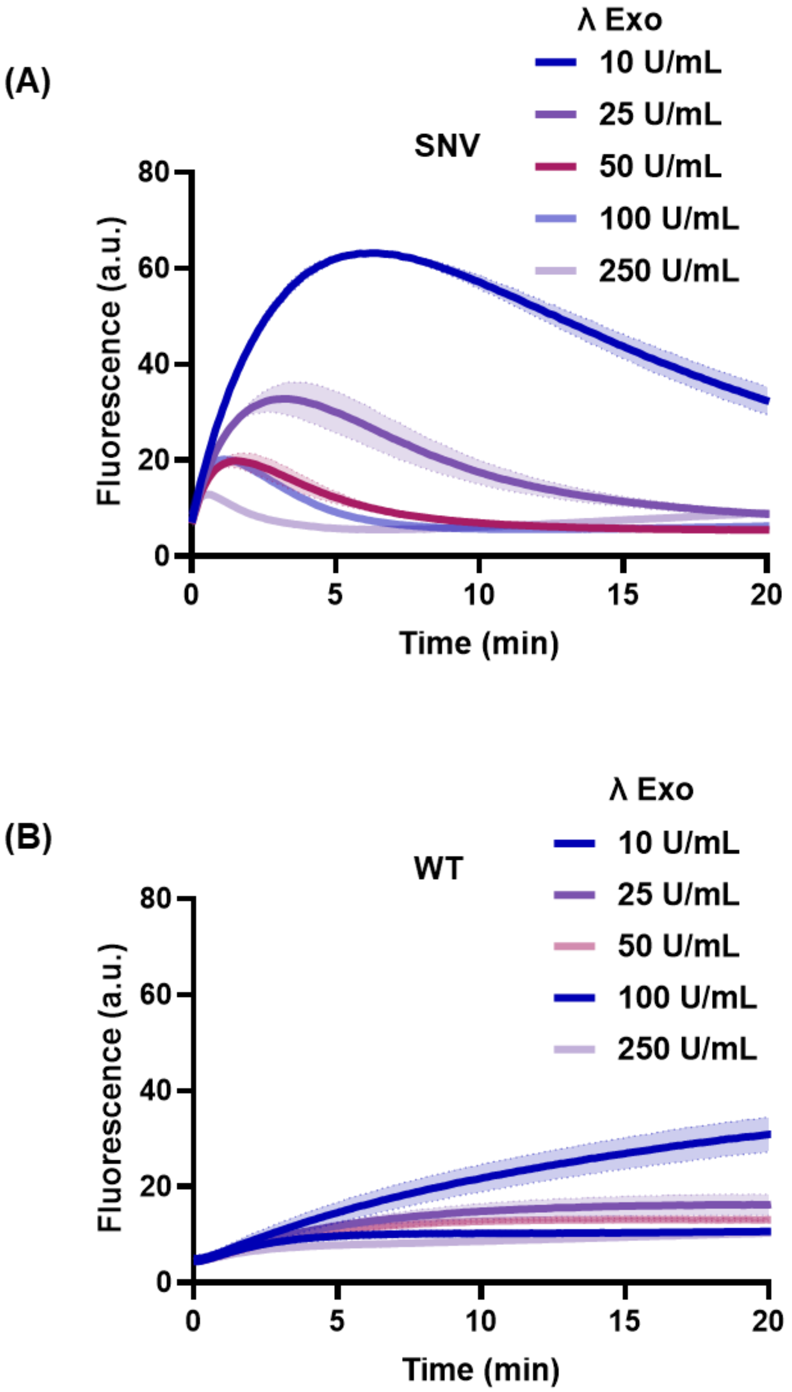

Figure S17. Exploring the effect of λ Exo concentration on DRPP (hairpin P-Probe) performance. (A) Fluorescence signals of SNV under different concentrations of λ Exo. (B) Fluorescence signals of WT under different concentrations of λ Exo. The forward toehold and reverse toehold of S-Probe were 7 and 5 nt, respectively. The mismatch site was located at the 7 nt of the 3' end of the forward toehold of S-Probe. All reactions were performed at 37 °C in 1×DRPP buffer. All nucleic acid species in DRPP were 1000 nM. Data are mean±S.D. (n = 3 independent experiments).

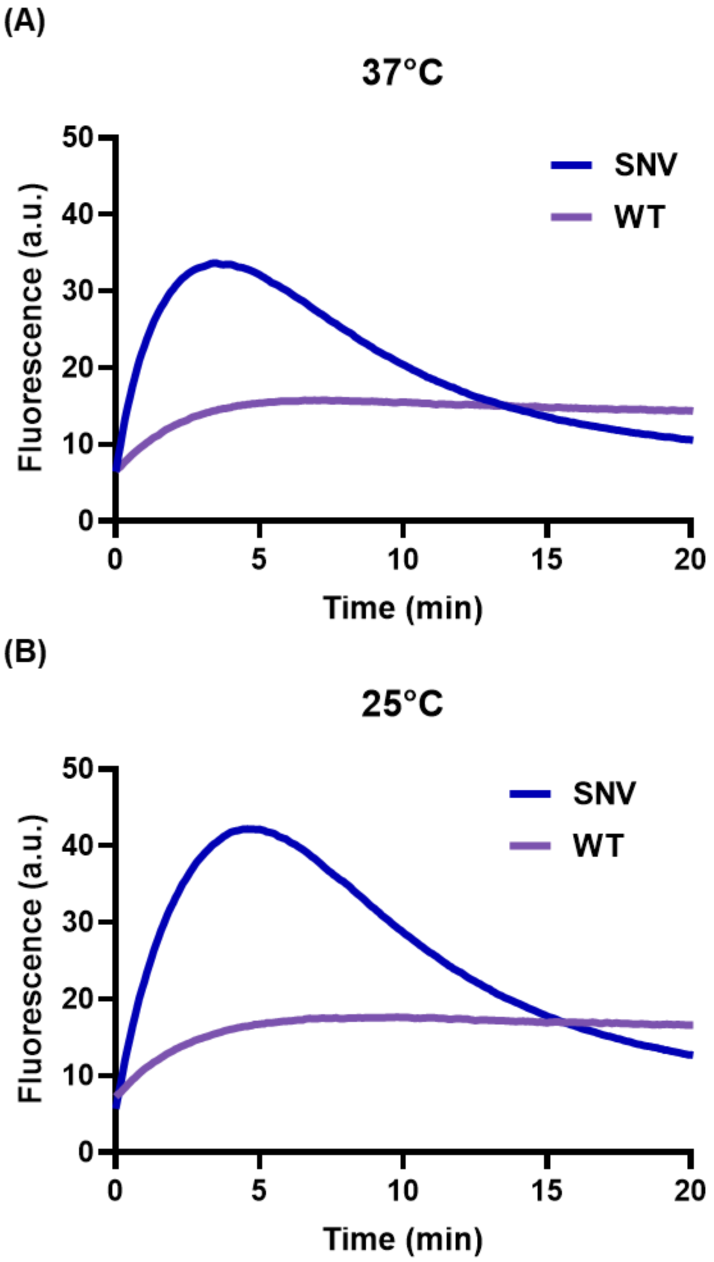

Figure S18. Exploration of the effect of reaction temperature on DRPP (hairpin P-Probe) performance. (A) DRPP assay for SNV and WT at 37 C°. (B) DRPP assay SNV and WT reactions at 25 C°. The forward toehold and reverse toehold of S-Probe were 7 and 5 nt, respectively. The mismatch site was located at the 7 nt of the 3' end of the forward toehold of S-Probe. All reactions were performed in a 1×DRPP buffer. All nucleic acid species in DRPP were 100 nM, and λ Exo was 50 U/mL.

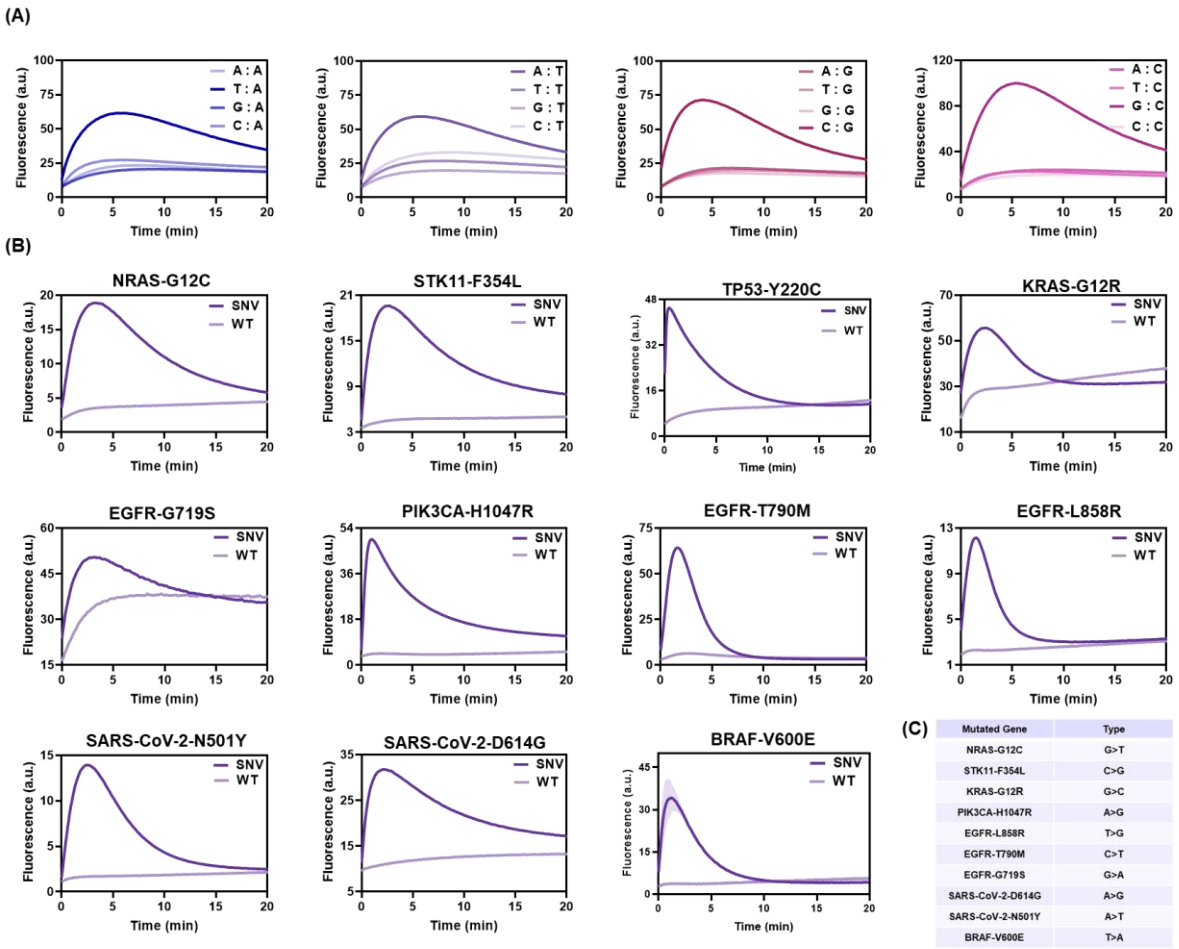

Mutated Gene	Type
NRAS-G12C	G>T
STK11-F354L	C>G
KRAS-G12R	G>C
PIK3CA-H1047R	A>G
EGFR-L858R	T>G
EGFR-T790M	C>T
EGFR-G719S	G>A
SARS-CoV-2-D614G	A>G
SARS-CoV-2-N501Y	A>T
BRAF-V600E	T>A

Figure S19. Identification of SNVs in different genes using DRPP (hairpin P-Probe). (A) DRPP signals for matches and mismatches. (B) Fluorescence kinetics of the 11 real genes from (C) detected by DRPP. (C) Mutation types of 11 disease-related genes, such as *KRAS-G12R* (G>C), indicating the gene mutation from G to T. The forward toehold and reverse toehold of S-Probe were 7 in DRPP were 100 nM, and λ Exo was 50 U/mL.

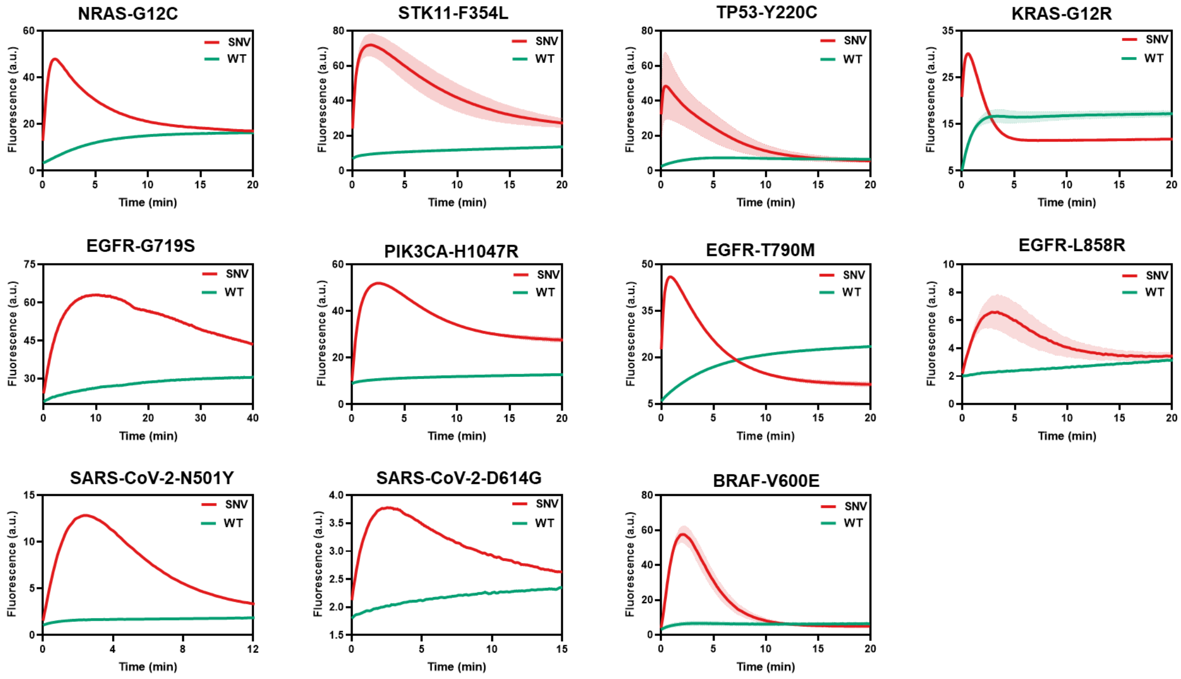

Figure S20. SNV detection in 11 genes by DRPP (double-stranded P-Probe). The forward toehold and reverse toehold of S-Probe were 7 and 5 nt, respectively. The mismatch site was located at the 7 nt of the 3' end of the forward toehold of S-Probe. All reactions were performed at 37 °C in 1×DRPP buffer. All nucleic acid species in DRPP were 100 nM, and λ Exo was 50 U/mL.

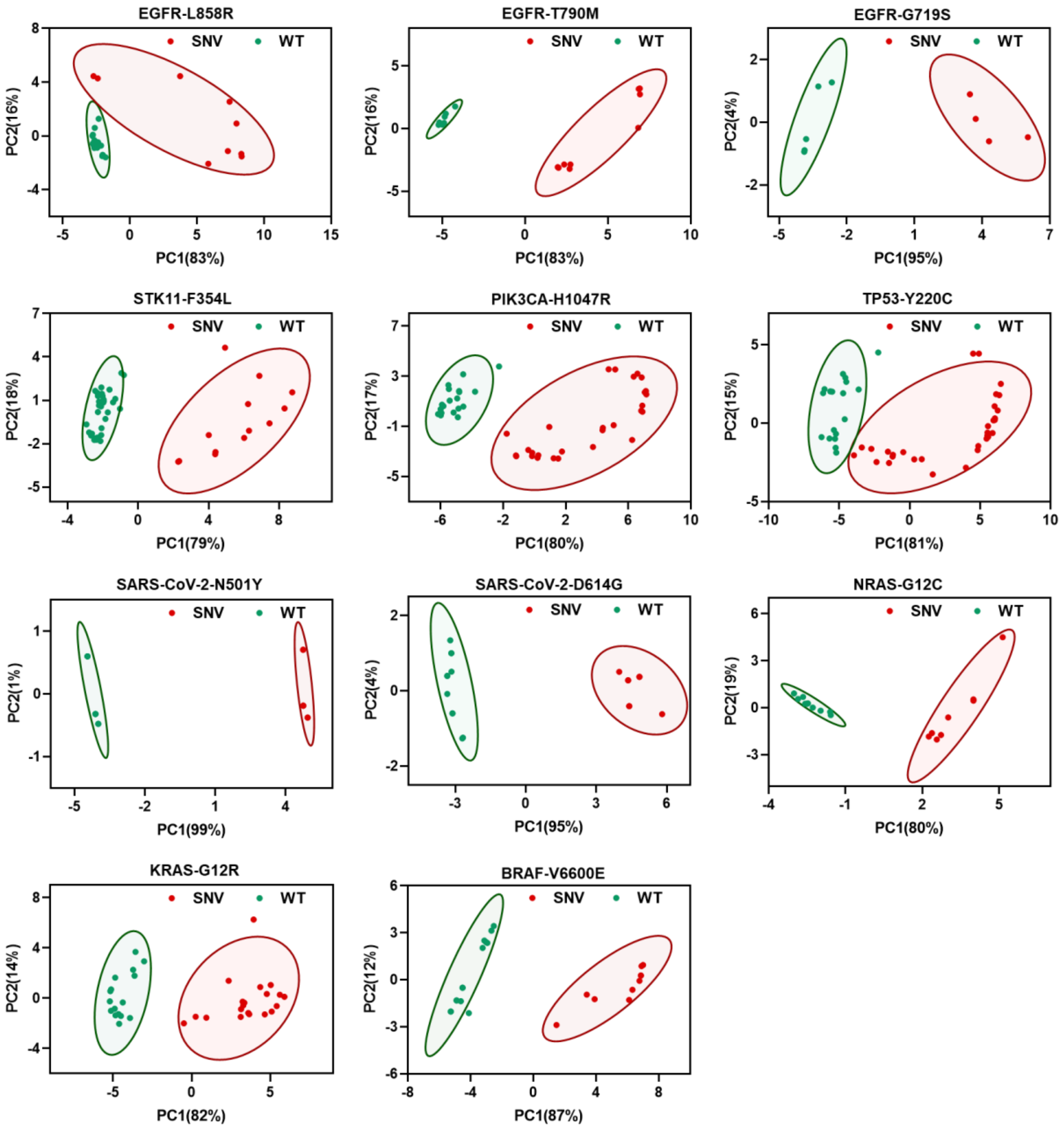

Figure S21. Principal component analysis of DRPP fluorescence kinetics of individual genes.

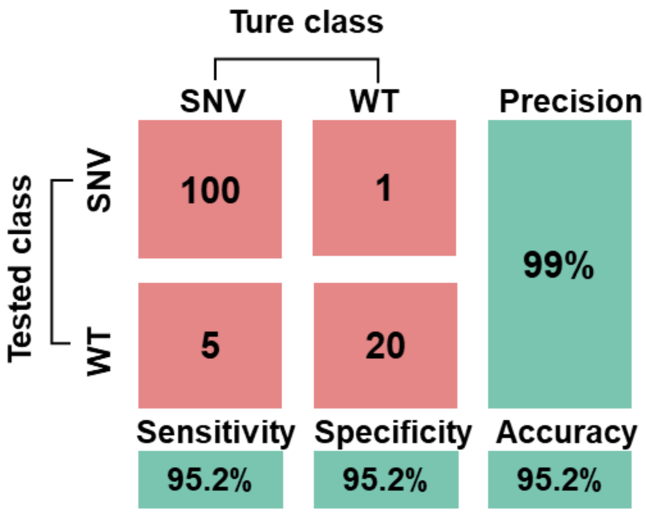

Figure S22. The binary classification confusion matrix for DRPP fluorescence kinetics of WT(VAF=0%) and SNV with various VAFs (VAF=0.1% (n=164), VAF=0.5% (n=111), VAF=2% (n=19), VAF=20% (n=19), and VAF=100% (n=35)) based on RF model.

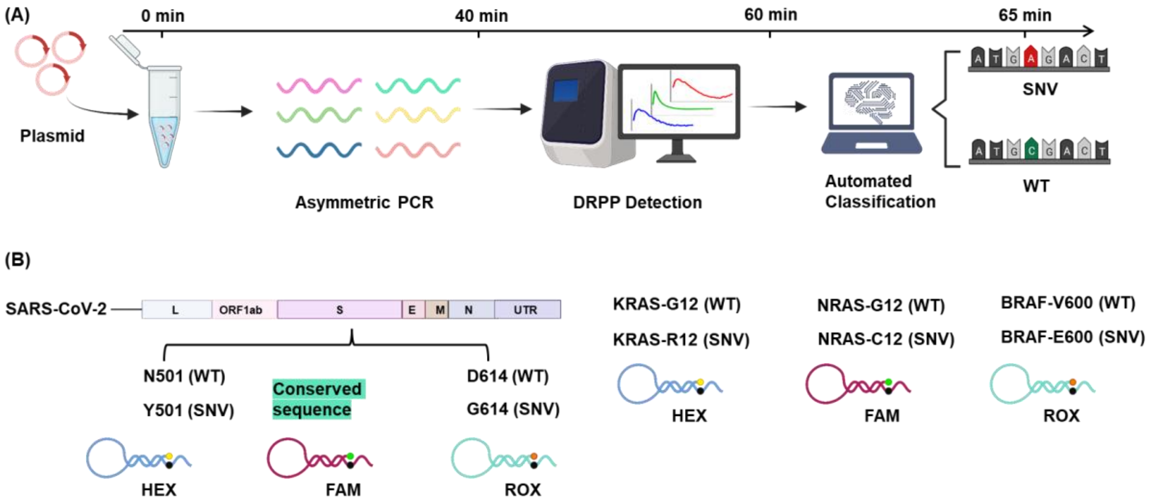

Figure S23. DRPP for the detection of SNVs in plasmid samples. (A) The workflow of the detection of SNV and WT in plasmid samples. Asymmetric PCR of plasmid samples generated single-stranded amplicons for DRPP assay, then the results of DRPP assay was used for machine learning-based automated classification. (B) The interested SNV sites and the corresponding P-Probe (multiplexed by dyes).

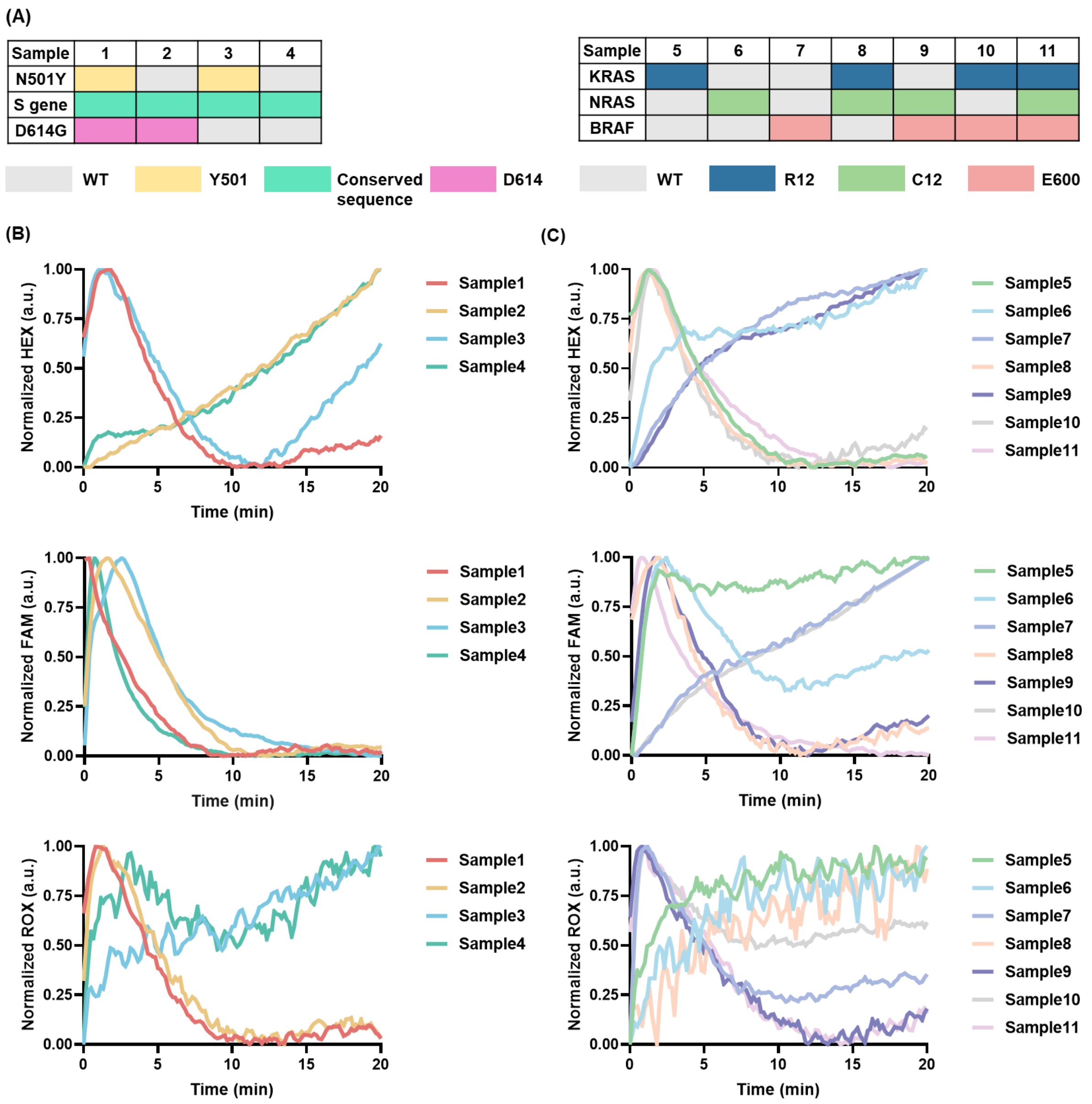

Figure S24. The DRPP fluorescence kinetics of the plasmid samples. (A) SNV information of the samples. (B) DRPP kinetics of all fluorescence channels of SARS-CoV-2 plasmid samples (#1-4), and (C) human gene plasmid samples (#5-11). The fluorescence intensity was normalized for the machine learning-based binary classification. The forward toehold and reverse toehold of S-Probe were 7 and 5 nt, respectively. The mismatch site was located at the 7 nt of the 3' end of the forward toehold of S-Probe. All reactions were performed at 37 °C in DRPP buffer. All nucleic acid species in DRPP were 100 nM, and λ Exo was 50 U/mL.

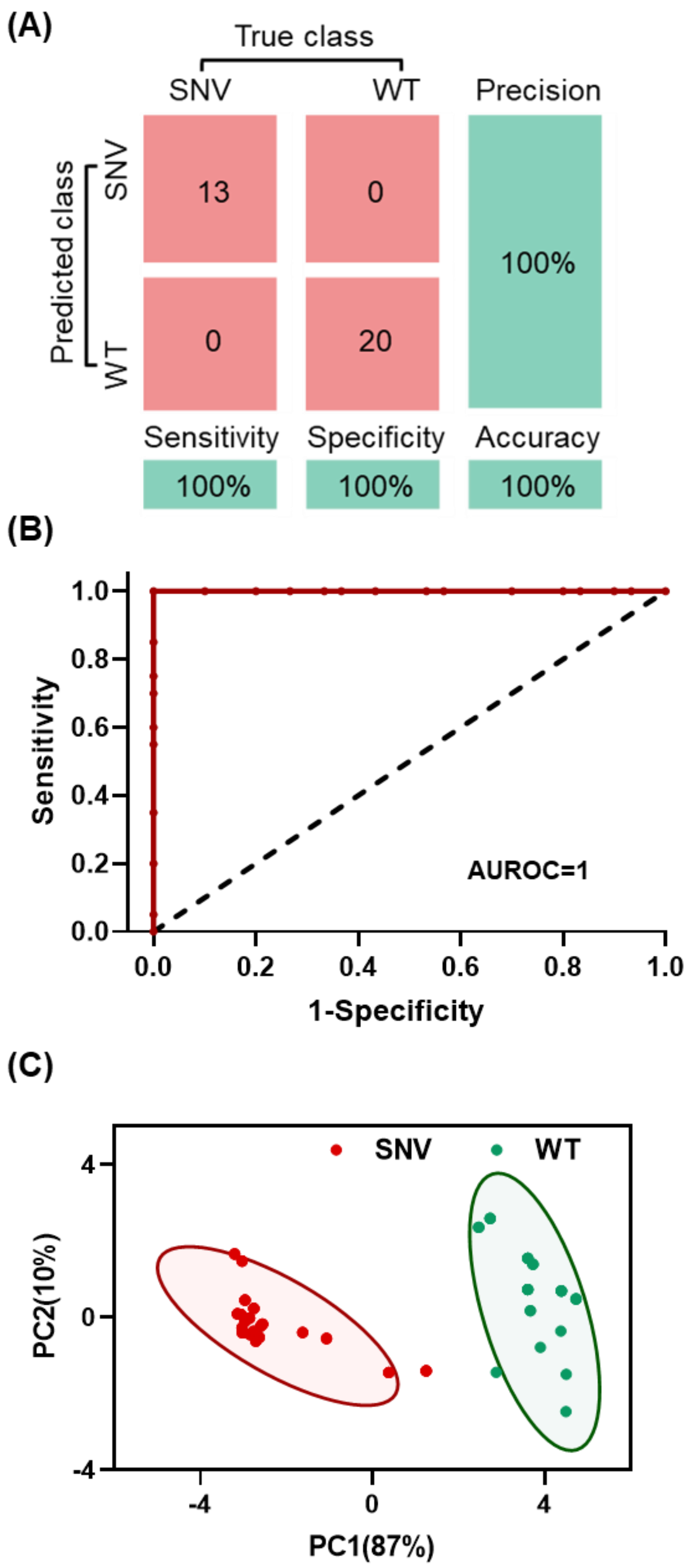

Figure S25. (A) The confusion matrix for the plasmid samples by RF classifier reveals 100% accuracy. (B) ROC curves and corresponding AUROC values. (C) Principal component analysis exhibited significant clustering of SNV and WT.

Supplementary Table

Table S1. Information of oropharyngeal swab samples of SARS-CoV-2 variants

No	Gender	Age	RT-qPCR C_t (orf1ab gene)	RT-qPCR C_t (N gene)	Variants
1	M	34	25.58	23.5	D614G/N501Y
2	M	28	20.96	23.16	D614G/N501Y
3	F	35	23.73	30.38	D614G/N501Y
4	F	45	21.83	25.37	D614G/N501Y
5	F	50	16.29	25.5	D614G/N501Y
6	F	46	19.08	29.61	D614G/N501Y
7	F	71	17.09	28.7	D614G/N501Y
8	M	50	18.12	24.81	D614G/N501Y
9	F	75	19.08	18.11	D614G/N501Y
10	M	57	22.09	19.08	D614G/N501Y
11	F	76	15.84	30.07	D614G/N501Y
12	F	33	30.35	15.48	D614G/N501Y
13	F	59	31.07	23.33	D614G/N501Y
14	F	36	23.34	17.85	D614G/N501Y
15	M	59	23.32	31.64	D614G/N501Y
16	M	60	20.74	27.12	D614G/N501Y
17	F	23	30.3	23.51	D614G/N501Y
18	F	34	21.28	23.01	D614G/N501Y
19	M	32	16.89	16.01	D614G/N501Y
20	M	59	28.27	26.6	D614G/N501Y
21	M	69	21.62	15.72	D614G/N501Y
22	F	39	19.11	16.21	D614G/N501Y

23	M	65	21.87	23.87	D614G/N501Y
24	F	59	16.64	16.64	D614G/N501Y
25	M	19	17.24	28.91	D614G/N501Y
26	F	55	31.02	28.9	D614G/N501Y
27	F	42	31.26	27.28	D614G/N501Y
28	F	73	24.78	17.54	D614G/N501Y
29	F	19	16.01	26.21	D614G/N501Y
30	F	46	18.99	23.82	D614G/N501Y
31	F	44	21	31.55	D614G/N501Y
32	M	46	28.96	26.03	D614G/N501Y
33	M	65	15.26	28.61	D614G/N501Y
34	F	38	15.73	22.71	D614G/N501Y
35	M	66	17.87	22.35	D614G/N501Y
36	F	47	26.04	29.03	D614G/N501Y
37	M	21	27.44	16.41	D614G/N501Y
38	M	29	26.01	17.26	D614G/N501Y
39	M	62	22.67	17.94	D614G/N501Y
40	M	47	24.3	21.64	D614G/N501Y
41	F	28	20.04	29.14	D614G/N501Y
42	F	39	27.66	28.66	D614G/N501Y
43	F	55	18.21	16.02	D614G/N501Y
44	M	30	26.68	21.79	D614G/N501Y
45	M	63	18.12	23.96	D614G/N501Y
46	F	78	27.09	23.19	D614G
47	M	32	18.77	17.05	D614G

48	M	25	16.99	25.02	D614G
49	F	25	20.04	18.84	D614G
50	M	22	20.42	21.54	D614G
51	F	43	22.21	24.91	D614G
52	F	45	23.63	19.28	D614G
53	F	40	16.45	19.94	D614G
54	M	64	19.46	25.49	D614G
55	M	56	28.62	19.51	D614G
56	F	65	15.49	29.02	D614G
57	M	74	30.79	31.71	D614G
58	F	77	27.42	27.42	D614G
59	F	30	23.31	20.84	D614G
60	F	27	24.84	24.93	D614G
61	M	54	15.64	16.6	N501Y
62	F	34	30.05	25.18	N501Y
63	M	21	30.53	23.01	N501Y
64	F	64	28.54	26.83	N501Y
65	F	33	16.67	26.9	N501Y
66	M	45	19.45	25.86	N501Y
67	M	60	20.7	15.57	N501Y
68	M	40	26.56	16.17	N501Y
69	M	63	17.32	20.43	N501Y
70	M	42	27.26	24.03	N501Y
71	M	60	16.81	26.13	N501Y
72	F	61	26.12	21.93	N501Y

73	F	45	23.4	28.94	N501Y
74	F	20	28.25	27.21	N501Y
75	M	38	27.16	31.47	N501Y
76	M	44	30.37	24.03	N501Y
77	F	35	30.15	20.53	N501Y
78	F	30	20.68	16.79	N501Y
79	F	68	26.88	25.39	N501Y
80	M	44	18.36	28.24	N501Y
81	F	72	15.51	22.2	N501Y
82	M	42	27.65	16.54	N501Y

Table S2. Information of oropharyngeal swab samples of SARS-CoV-2 wild type

No	Sex	Age	RT-qPCR Ct (orf1ab gene)	RT-qPCR Ct (N gene)
1	M	65	18.66	19.08
2	F	42	25.27	15.32
3	F	67	22.75	29.78
4	M	64	17.43	25.46
5	M	41	27.51	29.93
6	M	31	16.61	17.61
7	M	66	19.7	29.74
8	F	75	18.8	27.72
9	F	38	23.49	24.24
10	F	59	16.46	22.04
11	F	45	21.48	19.12
12	F	69	16.67	27.03

13	F	65	16.79	18.66
14	M	29	27.55	16.02
15	F	70	19.66	27.28
16	M	78	24.66	25.74
17	F	49	30.44	26.45
18	F	72	21.92	25.27
19	F	54	26.12	21.7
20	F	28	27.13	21.25
21	M	30	21.92	28.06
22	M	43	25.49	20.08
23	F	63	16.75	28.04
24	M	68	29.94	27.63
25	M	66	18	28.64
26	M	38	19.26	23.09
27	M	51	27.77	25.17
28	M	24	22.8	30.22
29	F	25	27.31	22.1
30	F	27	21.34	15.96
31	F	59	19.36	28.87
32	F	48	15.59	25.1
33	F	30	25.77	20.68
34	F	48	21.87	30.96
35	M	27	22.23	18.58
36	F	22	24.76	25.44
37	M	70	15.95	24.68

38	F	52	20.05	21.19
39	F	74	27.37	17.27
40	F	60	26.14	15.4
41	F	53	17	21.74
42	M	67	17.08	17.94
43	M	71	16.47	26.61
44	F	78	15.12	20.92
45	M	19	21.77	28.47
46	M	70	25.49	26.75
47	M	55	26.57	24.14

Table S3 Clinicopathological information for 141 ovarian cancer patients from whom cancer/normal tissue samples were collected.

No	Pathological Type	Stage	Recurrence
1	High-grade serous adenocarcinoma	IIIC	No
2	High-grade serous adenocarcinoma	IVB	No
3	High-grade serous adenocarcinoma	IIIC	No
4	Clear cell carcinoma	IIIC	No
5	High-grade serous adenocarcinoma	-	Yes
6	High-grade serous adenocarcinoma	IIIC	No
7	High-grade serous adenocarcinoma	IIIA	No
8	High-grade serous adenocarcinoma	IIIC	No
9	Endometrioid carcinoma	IIIC	No
10	High-grade serous adenocarcinoma	IIIA	No
11	High-grade serous adenocarcinoma	IIIC	No
12	High-grade serous adenocarcinoma	IIIA	No

13	High-grade serous adenocarcinoma	IIIC	No
14	High-grade serous adenocarcinoma	IIIC	No
15	Endometrioid carcinoma	IIIC	No
16	High-grade serous adenocarcinoma	IIIC	No
17	Clear cell carcinoma	IIIA	No
18	High-grade serous adenocarcinoma	IIIA	No
19	High-grade serous adenocarcinoma	IIIC	No
20	Endometrioid carcinoma	IIIC	No
21	High-grade serous adenocarcinoma	IIIC	No
22	High-grade serous adenocarcinoma	IIIC	No
23	High-grade serous adenocarcinoma	IIIC	No
24	High-grade serous adenocarcinoma	-	Yes
25	High-grade serous adenocarcinoma	IIIC	No
26	Clear cell carcinoma	IIIC	No
27	High-grade serous adenocarcinoma	IIIC	No
28	High-grade serous adenocarcinoma	IIIC	No
29	High-grade serous adenocarcinoma	IIIA	No
30	High-grade serous adenocarcinoma	IIIA	No
31	High-grade serous adenocarcinoma	IIIC	No
32	High-grade serous adenocarcinoma	IIIC	No
33	Poorly differentiated endometrioid carcinoma	IC	No
34	Clear cell carcinoma	IIIC	No
35	High-grade serous adenocarcinoma	IIIC	No
36	High-grade serous adenocarcinoma	IIIA	No
37	High-grade serous adenocarcinoma	IIIC	No

38	High-grade serous adenocarcinoma	IIIA	No
39	High-grade serous adenocarcinoma	IIIC	No
40	High-grade serous adenocarcinoma	IIIC	No
41	High-grade serous adenocarcinoma	IIIA	No
42	High-grade serous adenocarcinoma	IIIC	No
43	Poorly differentiated endometrioid carcinoma	IC	No
44	High-grade serous adenocarcinoma	IIIC	No
45	High-grade serous adenocarcinoma	IIIA	No
46	High-grade serous adenocarcinoma	IIIA	No
47	High-grade serous adenocarcinoma	IIIC	No
48	Poorly differentiated endometrioid carcinoma	IIIC	No
49	High-grade serous adenocarcinoma	IIIC	No
50	High-grade serous adenocarcinoma	-	Yes
51	High-grade serous adenocarcinoma	IIIC	No
52	High-grade serous adenocarcinoma	IIIC	No
53	High-grade serous adenocarcinoma	IIIC	No
54	High-grade serous adenocarcinoma	IC	No
55	High-grade serous adenocarcinoma	IIIA	No
56	Poorly differentiated endometrioid carcinoma	IIIC	No
57	High-grade serous adenocarcinoma	IIIC	No
58	Poorly differentiated endometrioid carcinoma	IC	No
59	High-grade serous adenocarcinoma	IIIC	No
60	High-grade serous adenocarcinoma	IVB	No
61	High-grade serous adenocarcinoma	IIIC	No
62	High-grade serous adenocarcinoma	IIIC	No

63	Endometrioid carcinoma	IIIC	No
64	High-grade serous adenocarcinoma	IIIC	No
65	High-grade serous adenocarcinoma	IIIC	No
66	Poorly differentiated endometrioid carcinoma	IIIA	No
67	High-grade serous adenocarcinoma	IIIC	No
68	High-grade serous adenocarcinoma	IIIC	No
69	High-grade serous adenocarcinoma	IIIC	No
70	High-grade serous adenocarcinoma	IIIC	No
71	High-grade serous adenocarcinoma	IIIC	No
72	High-grade serous adenocarcinoma	IIIA	No
73	High-grade serous adenocarcinoma	IIIA	No
74	High-grade serous adenocarcinoma	IIIA	No
75	High-grade serous adenocarcinoma	IIIA	No
76	High-grade serous adenocarcinoma	IIIC	No
77	High-grade serous adenocarcinoma	IIIA	No

Table S4. The oligonucleotide sequence of SRPP.

	Species	Sequence（5`-3`）
SRPP	SNV	AAACTGGTGGTGGTTGGAGCATGTGGTG
	WT	AAACTGGTGGTGGTTGGAGCAGGTGGTG
	TMSD-Probe1	ACACCTTTAACCCGTACCATTTTTTAAACTGGTGGTGGTTGGAGCA
	TMSD-Probe2	CACCACATGCTCCAACCACCACCAGTTTGTTCCGGAGTGTAAGTATGAATTTCCAGGACG
	TMSD-Probe3	5`-BHQ1-AAAAAATGGTACGGGTTAAAGGTGT
	TMSD-Probe4	CGTCCTGGAAATTCATACTTACACTCCGGAAC-FAM-`3

Table S5. The oligonucleotide sequence of DRPP.

Mutation	Species	Sequence（5`-3`）
KRAS-G12R (G>C)	SNV	AAACTTGTGGTAGTTGGAGCTCGTGGCG
	WT	AAACTTGTGGTAGTTGGAGCTGGTGGCG
	S-Probe1	TTTGTCTTTGTATTTGTCTGG
	Messenger DNA	5`-P-GTATGAAACTTGTGGTAGTTGGAGCT
	S-Probe2	CGCCACGAGCTCCAACTACCACAAGTTTCA TACCCAGACAAATACAAAGACAAA
	P-Probe (hairpin)	5`-HEX- ACTTGTGGTAGTTGGAGCTACTCACCCTCA ACCTCAGCTCCAACTACCACAAG/dT- BHQ1/TTTCATAC
	P-Probe1 (double-strand)	TTACTCACCCTCAACCTCAGCTCCAACTACC ACAAGTTTCATAC
	P-Probe2 (double-strand)	ACTTGTGGTAGTTGGAGCT-HEX-`3
	P-Probe3 (double-strand)	5`-BHQ1-GAGGTTGAGGGTGAGTAA
	SNV (fluorophore FAM)	AAACTTGTGGTAGTTGGAGC/dT- FAM/TCGTGGCG
	WT (fluorophore FAM)	AAACTTGTGGTAGTTGGAGC/dT- FAM/TGGTGGCG
	P-Probe (Quencher BHQ1)	ACTTGTGGTAGTTGGAGCTACTCACCCTCA ACCTCAGC/dT- BHQ1/TCCAACTACCACAAGTTTCATAC
	S-Probe2 (7nt forward toehold)	CGCCACGAGCTCCAACTACCACAAGTTTCA TACCCAGACAAATACAAAGACAAA
	S-Probe2 (6nt forward toehold)	GCCACGAGCTCCAACTACCACAAGTTTCAT ACCCAGACAAATACAAAGACAAA
	S-Probe2 (5nt forward toehold)	CCACGAGCTCCAACTACCACAAGTTTCATA CCCAGACAAATACAAAGACAAA
	SNV (7 nt reverse toehold)	AAACTTGTGGTAGTTGGAGCTCGTGGCG
	WT (7 nt reverse toehold)	AAACTTGTGGTAGTTGGAGCTGGTGGCG
	SNV	AACTTGTGGTAGTTGGAGCTCGTGGCG

	(6 nt reverse toehold)	
	WT<br>(6 nt reverse toehold)	AACTTGTGGTAGTTGGAGCTGGTGGCG
	SNV<br>(5 nt reverse toehold)	ACTTGTGGTAGTTGGAGCTCGTGGCG
	WT<br>(5 nt reverse toehold)	ACTTGTGGTAGTTGGAGCTGGTGGCG
	A (Target)	AAACTTGTGGTAGTTGGAGCTAGTGGCG
	T (Target)	AAACTTGTGGTAGTTGGAGCTTGTGGCG
	C (Target)	AAACTTGTGGTAGTTGGAGCTCGTGGCG
	G (Target)	AAACTTGTGGTAGTTGGAGCTGGTGGCG
	A (Probe)	CGCCACAAGCTCCAACTACCACAAGTTTCATACCCAGACAAATACAAAGACAAA
	T (Probe)	CGCCACTAGCTCCAACTACCACAAGTTTCATACCCAGACAAATACAAAGACAAA
	C (Probe)	CGCCACCAGCTCCAACTACCACAAGTTTCATACCCAGACAAATACAAAGACAAA
	G (Probe)	CGCCACGAGCTCCAACTACCACAAGTTTCATACCCAGACAAATACAAAGACAAA
	Match	AAACTTGTGGTAGTTGGAGCTCGTGGCG
	Mismatch1	AAACTTGTGGTAGTTGGAGCTCGTCGCG
	Mismatch2	AAACTTGTGGTAGTTGGAGCTCCTGGCG
	Mismatch3	AAACTTGTGGTAGTTGGAGCTGGTGGCG
	Mismatch4	AAACTTGTGGTAGTTGGACCTCGTGGCG
PIK3CA-H1047R (A>G)	SNV	TGAAACAAATGAATGATGCACGTCATGG
	WT	TGAAACAAATGAATGATGCACATCATGG
	S-Probe1	CTTAGCATCGAGTCCAGTCCA
	Messenger DNA	5`-P-GGACTTGAAACAAATGAATGATGCAC
	S-Probe2	CCATGACGTGCATCATTCATTTGTTTCAAGTCCTGGACTGGACTCGATGCTAAG
	P-Probe<br>(hairpin)	5`-FAM-AAACAAATGAATGATGCACATGACCTGAACTGAGCGTGCATCATTCATTTGTT/dT-BHQ1/TCAAGTCC
	P-Probe1<br>(double-strand)	CAGACTACAGTACTGCCTGTGCATCATTCATTTGTTTCAAGTCC

	P-Probe2 (double-strand)	AAACAAATGAATGATGCAC-FAM-`3
	P-Probe3 (double-strand)	5`-BHQ1-AGGCAGTACTGTAGTCTG
BRAF-V600E (T>A)	SNV	GTGATTTTGGTCTAGCTACAGAGAAATC
	WT	GTGATTTTGGTCTAGCTACAGTGAAATC
	S-Probe1	CAGCGCCCGTCTGTTGGCCGG
	Messenger DNA	GAACGGTGATTTTGGTCTAGCTACAG
	S-Probe2	GATTTCTCTGTAGCTAGACCAAAATCACCG TTCCCGGCCAACAGACGGGCGCTG
	P-Probe (hairpin)	5`-FAM- GATTTTGGTCTAGCTACAGCGTTTCTTCAAT ATTACTGTAGCTAGACCAAAA/dT- BHQ1/TCACCGTTC
	P-Probe1 (double-strand)	TTCTCCATTATCCACCTCCTGTAGCTAGACC AAAATCACCGTTC
	P-Probe2 (double-strand)	GATTTTGGTCTAGCTACAG-FAM-`3
	P-Probe3 (double-strand)	5`-BHQ1-GAGGTGGATAATGGAGAA
NRAS-G12C (G>T)	SNV	AAACTGGTGGTGGTTGGAGCATGTGGTG
	WT	AAACTGGTGGTGGTTGGAGCAGGTGGTG
	S-Probe1	GGGATGTGAGGATTGAGGGCG
	Messenger DNA	5`-P-ATTGGAAACTGGTGGTGGTTGGAGCA
	S-Probe2	CACCACATGCTCCAACCACCACCAGTTTCC AATCGCCCTCAATCCTCACATCCC
	P-Probe (hairpin)	5`-FAM- ACTGGTGGTGGTTGGAGCACACTTCACACT CCCACTGCTCCAACCACCACCAG/dT- BHQ1/TTTCATCC
	P-Probe1 (double-strand)	CACCTCACCTCCACCTTACCCTGCTCCAACC ACCACCAGTTTCCAAT
	P-Probe2 (double-strand)	ACTGGTGGTGGTTGGAGCA-FAM-`3
	P-Probe3 (double-strand)	5`-BHQ1-GGGTAAGGTGGAGGTGAGGTG

STK11-5354L (C>G)	SNV	GGACGAGGACGAGGACCTCTTGGACATC
	WT	GGACGAGGACGAGGACCTCTTCGACATC
	S-Probe1	TGTTTAGGCGGTGTTATCTGA
	Messenger DNA	5`-P-TGAGTGGACGAGGACGAGGACCTCTT
	S-Probe2	GATGTCCAAGAGGTCCTCGTCCTCGTCCACT CATCAGATAACACCGCCTAAACA
	P-Probe (hairpin)	5`-FAM- ACGAGGACGAGGACCTCTTGACTACCGAAC TTACTAAGAGGTCCTCGTCCTCG/dT- BHQ1/TCCACTCA
	P-Probe1 (double-strand)	TCATCGATGATGCGTATGAAGAGGTCCTCG TCCTCGTCCACTCA
	P-Probe2 (double-strand)	ACGAGGACGAGGACCTCTT-FAM-`3
	P-Probe3 (double-strand)	5`-BHQ1-CATACGCATCATCGATGA
TP53-Y220C (A>G)	SNV	GACATAGTGTGGTGGTGCCCTGTGAGCC
	WT	GACATAGTGTGGTGGTGCCCTATGAGCC
	S-Probe1	CTCGAATCAGACTAAGCATAC
	Messenger DNA	5`-P-AACGGGACATAGTGTGGTGGTGCCCT
	S-Probe2	GGCTCACAGGGCACCACCACACTATGTCCC GTTGTATGCTTAGTCTGATTCGAG
	P-Probe (hairpin)	5`-FAM- CATAGTGTGGTGGTGCCCTAGTCTATGCCAT GTTCAGGGCACCACCACACTATG/dT- BHQ1/TCCCGTT
	P-Probe1 (double-strand)	GGCTGGCACTAGCGTCTGAGGGCACCACCA CACTATGTCCCGTT
	P-Probe2 (double-strand)	CATAGTGTGGTGGTGCCCT-FAM-`3
	P-Probe3 (double-strand)	5`-BHQ1-CAGACGCTAGTGCCAGCC
EGFR-L858R (T>G)	SNV	TCAAGATCACAGATTTTGGGCGGGCCAA
	WT	TCAAGATCACAGATTTTGGGCTGGCCAA
	S-Probe1	GGAGTAGGTAAGAGTAAGTAG
	Messenger DNA	5`-P-GGTAGTCAAGATCACAGATTTTGGGC

	S-Probe2	TTGGCCCGCCCAAAATCTGTGATCTTGACTA CCCTACTTACTCTTACCTACTCC
	P-Probe (hairpin)	5`-FAM-AAGATCACAGATTTTGGGCCTCCATTATCC ACCTCGCCCAAAATCTGTGATCT/dTBHQ1/T GACTACC
	P-Probe1 (double-strand)	TTCTCCATTATCCACCTCGCCCAAAATCTGT GATCTTGACTACC
	P-Probe2 (double-strand)	AAGATCACAGATTTTGGGC-FAM`3-
	P-Probe3 (double-strand)	5`-BHQ1-GAGGTGGATAATGGAGAA
EGFR-T790M (C>T)	SNV	CCTCCACCGTGCAGCTCATCATGCAGCT
	WT	CCTCCACCGTGCAGCTCATCACGCAGCT
	S-Probe1	ACAACTCACCTCAACTAAACT
	Messenger DNA	5`-P-CTAAACCTCCACCGTGCAGCTCATCA
	S-Probe2	AGCTGCATGATGAGCTGCACGGTGGAGGTT TAGAGTTTAGTTGAGGTGAGTTGT
	P-Probe (hairpin)	5`-FAM-TCCACCGTGCAGCTCATCATATCACCTCCAA CTCTTGATGAGCTGCACGG/dTBHQ1/TGGAG GTTTAG
	P-Probe1 (double-strand)	CCTATCACCTCCAACTCTTGATGAGCTGCAC GGTGGAGGTTTAG
	P-Probe2 (double-strand)	TCCACCGTGCAGCTCATCA-FAM-`3
	P-Probe3 (double-strand)	5`-BHQ1-AGAGTTGGAGGTGATAGG
EGFR-G719S (A>G)	SNV	TTCAAAAAGATCAAAGTGCTGAGCTCCG
	WT	TTCAAAAAGATCAAAGTGCTGGGCTCCG
	S-Probe1	CCTGATCGACTCGGACTCAGT
	Messenger DNA	5`-P-CATAGTTCAAAAAGATCAAAGTGCTG
	S-Probe2	CGGAGCTCAGCACTTTGATCTTTTTGAACTA TGACTGAGTCCGAG TCGATCAGG
	P-Probe (hairpin)	5`-FAM-CAAAAAGATCAAAGTGCTGCTCAGGCTAGA

		CGTTACAGCACTTTGATCTTTT/dT-BHQ1/TGAACTATG
	P-Probe1 (double-strand)	CCTATCACCTCCAACTCTCAGCACTTTGATCTTTTTGAACTATG
	P-Probe2 (double-strand)	CAAAAAGATCAAAGTGCTG-HEX-`3
	P-Probe3 (double-strand)	5`-BHQ1-AGAGTTGGAGGTGATAGG
SARS-CoV-2-D614G (A>G)	SNV	AGGTTGCGGTGCTGTATCAAGGCGTTAA
	WT	AGGTTGCGGTGCTGTATCAAGACGTTAA
	S-Probe1	ACTTGACGATTCAGACTGACC
	Messenger DNA	5`-P-GACGTAGGTTGCGGTGCTGTATCAAG
	S-Probe2	TTAACGCCTTGATACAGCACCGCAACCTACGTCGGTCAGTCTGAATCGTCAAGT
	P-Probe (hairpin)	5`-ROX-GTTGCGGTGCTGTATCAAGATGTACAAGTGCTAAACTTGATACAGCACCGCAACC/dT-BHQ1/TACGTC
	P-Probe1 (double-strand)	CCTATCACCTCCAACTCTGGTCGGTTGGAAACCGTAGGCTCGAT
	P-Probe2 (double-strand)	GTTGCGGTGCTGTATCAAG-3`ROX
	P-Probe3 (double-strand)	5`-BHQ1-AGAGTTGGAGGTGATAGG
SARS-CoV-2-N501Y (A>G)	SNV	AGCTACGGTTTCCAACCGACCTACGGTG
	WT	AGCTACGGTTTCCAACCGACCAACGGTG
	S-Probe1	CATCGCTAGTTCGACATCGGC
	Messenger DNA	5`-P-CATCGAGCTACGGTTTCCAACCGACC
	S-Probe2	CACCGTAGGTCGGTTGGAAACCGTAGCTCGATGCCGATGTCGAACTAGCGATG
	P-Probe (hairpin)	5`-HEX-CTACGGTTTCCAACCGACCAATGACTGAAGCTGAAGGTCGGTTGGAAACCG/dT-BHQ1/TAGGCTCGAT
	P-Probe1 (double-strand)	CCTATCACCTCCAACTCTCAGCACTTTGATCTTTTTGAACTATG

	P-Probe2 (double-strand)	CTACGGTTTCCAACCGACC-3`HEX
	P-Probe3 (double-strand)	5`-BHQ1-AGAGTTGGAGGTGATAGG
SARS-CoV-2-S gene conserved sequence	SNV	CAGCAATTCGGTCGTGACATCGCGGATA
	WT	CTTTGACGATTCAGTCGAGCT
	S-Probe1	5`-P-ATGACCAGCAATTCGGTCGTGACATC
	Messenger DNA	TATCCGCGATGTCACGACCGAATTGCTGGT CATAGCTCGACTGAATCGTCAAAG
	S-Probe2	5`-FAM-GCAATTCGGTCGTGACATCCAATCGTACAG TCAAAGATGTCACGACCGAATTGC/dT-BHQ1/TGGTCAT
	P-Probe (hairpin)	CCTATCACCTCCAACTCTGATGTCACGACCG AATTGCTGGTCAT
	P-Probe1 (double-strand)	GTTGCGGTGCTGTATCAAG -FAM-`3
	P-Probe2 (double-strand)	5`-BHQ1-AGAGTTGGAGGTGATAGG

Table S6 The primer sequence of asymmetric PCR used in this work.

	Species	Sequence（5`-3`）
KRAS-G12R	Forward Primer	GGCCTGCTGAAAATGACTGAATA
	Reverse Primer	TAGCTGTATCGTCAAGGCAC
NRAS-G12C	Forward Primer	TCCAACAGGTTCTTGCTGGT
	Reverse Primer	ATTGTCAGTGCGCTTTTCCC
BRAF-V600E	Forward Primer	AGACCTCACAGTAAAAATAGGTGATT
	Reverse Primer	TCAAACTGATGGGACCCACTC
SARS-CoV-2-N501Y	Forward Primer	GCAGTTCAAAGCTCAGCACA
	Reverse Primer	GCTGCAAAGCTACGGTTTCC
SARS-CoV-2-S gene conserved sequence	Forward Primer	CCGAGAGCAACAAGAAATTCCT
	Reverse Primer	CCAGGGTCTGCGGGTCAC
SARS-CoV-2-D614G	Forward Primer	GGTGGCGTTAGCGTGATCA
	Reverse Primer	CCGGAACTTCGGTGCAGTTA

Reference

[1] aJ. Liu, Y. Liu, L. Zhang, S. Fu, X. Su, *Biosens. Bioelectron.* **2022**, *215*, 114561; bN. Li, Y. Zhao, Y. Liu, Z. Yin, R. Liu, L. Zhang, L. Ma, X. Dai, D. Zhou, X. Su, *Nano Today* **2021**, *41*, 101308; cA. M. Van Oijen, P. C. Blainey, D. J. Crampton, C. C. Richardson, T. Ellenberger, X. S. Xie, *Science* **2003**, *301*, 1235-1238.

[2] J. N. Zadeh, C. D. Steenberg, J. S. Bois, B. R. Wolfe, M. B. Pierce, A. R. Khan, R. M. Dirks, N. A. Pierce, *J. Comput. Chem.* **2011**, *32*, 170-173.
